# Phosphorylation disrupts the interaction between the intrinsically disordered region of the oncogenic NDRG1 and lipid vesicles

**DOI:** 10.1002/pro.70510

**Published:** 2026-02-22

**Authors:** Noemi Carosella, Chiara Pastorello, Jehan Waeytens, Ylenia Beniamino, Valentina Roncassaglia, Lucrezia Serra, Vincent Raussens, Elisabetta Mileo, Stefano Ciurli, Barbara Zambelli

**Affiliations:** ^1^ Laboratory of Bioinorganic Chemistry, Department of Pharmacy and Biotechnology (FaBiT) University of Bologna Bologna Italy; ^2^ Aix‐Marseille Univ, CNRS, BIP, Bioénergétique et Ingénierie des Protéines Marseille France; ^3^ Aix‐Marseille Univ, CNRS, INSERM, Institut Paoli‐Calmettes, CRCM Marseille France; ^4^ RD3‐Pharmacognosy, Bioanalysis and Drug Discovery Unit, Faculty of Pharmacy Université Libre de Bruxelles Bruxelles Belgium; ^5^ Structure et Fonction des Membranes Biologiques Université libre de Bruxelles Bruxelles Belgium; ^6^ Present address: IRCCS Istituto delle Scienze Neurologiche di Bologna Bologna Italy

**Keywords:** intrinsically disordered region, lung cancer, membrane binding, NDRG1, nickel‐driven carcinogenesis

## Abstract

NDRG1 is a multifunctional regulatory human protein implicated in crucial cellular processes and acting as a central hub of a cancer‐related interactome. In lung cancer, increased NDRG1 expression is associated with resistance to chemotherapy and is linked to the cellular response to nickel, a known tumor driver in air pollution. Although NDRG1 has been extensively studied in relation to cancer and lipid vesicle recycling, its precise molecular mechanisms remain poorly defined. To address this gap, the present work shifts the focus from previous cellular‐level investigations into a molecular‐level study of NDRG1, dissecting the effects of phosphorylation, metal binding, and lipid interactions. Specifically, we investigated the C‐terminal intrinsically disordered region of the protein that is a key regulatory hub and an attractive target for drug development. Employing a multimodal approach, which combines circular dichroism (CD), nuclear magnetic resonance (NMR), electron paramagnetic resonance (EPR), Fourier‐transform infrared spectroscopy (FT‐IR), and isothermal titration calorimetry (ITC), we developed a mechanistic rationale for how phosphorylation modulates protein subcellular localization, lipid trafficking and storage, acting as a molecular switch that toggles NDRG1 between membrane‐bound, adhesion‐supporting states and soluble, signaling‐competent conformations with metal‐binding activity. These insights open potential opportunities for therapeutic intervention in nickel and pollution‐driven lung cancer.

## 1 INTRODUCTION

N‐myc downstream‐regulated gene 1 (NDRG1) is a widely expressed and multifunctional protein involved in crucial cellular processes, including growth, differentiation, proliferation, lipid recycling, and stress responses (Fang et al. 2014). Many of its functions are linked to cancer biology, where NDRG1 exhibits pleiotropic and context‐dependent effects that vary across tumor types, reflecting its interplay with several oncogenes and onco‐suppressor genes (Kitowska and Pawełczyk 2010). In some cancers, such as brain, colorectal, ovarian, and prostate malignancies, NDRG1 acts as a metastasis suppressor limiting tumor progression. Conversely, in cervical, hepatocellular, renal, gastric, and lung cancers, it functions as an oncogene (Ghafouri‐Fard et al. 2023; Joshi et al. 2022). In breast cancer, NDRG1 exerts a dual role: initially considered a metastasis suppressor, it has more recently been identified as a pro‐oncogenic and pro‐metastatic effector (Villodre et al. 2024). This duality underscores its functional versatility and shows that dysregulation of NDRG1 is a hallmark of poor prognosis and cancer aggressiveness. Furthermore, NDRG1 expression levels correlate with increasing resistance or sensitization of tumor cells to chemotherapy or radiotherapy (Du et al. 2018; Joshi et al. 2022; Motwani et al. 2002). Collectively, these findings have positioned NDRG1 as a critical therapeutic target.

Understanding the mechanisms underlying NDRG1 function and integration into signaling cascades related to cancer is therefore essential to design targeted therapies that modulate protein functions. However, despite extensive research, the molecular details of its role remain largely unknown. NDRG1 participates in multiple signaling pathways governing cell growth and differentiation (Park et al. 2020) and interacts with a wide range of proteins, acting as a central hub of a large interactome (Beniamino et al. 2022). It also displays broad subcellular distribution: although predominantly cytoplasmic, it has been detected in nuclear, mitochondrial, and membrane compartments, despite lacking canonical targeting sequences (Kitowska and Pawełczyk 2010).

In lung cancer, the leading cause of cancer‐related mortality worldwide, NDRG1 overexpression is consistently associated with tumor aggressiveness. High NDRG1 levels represent a negative prognostic marker, correlating with tumor progression through epithelial‐to‐mesenchymal transition and acquisition of stem‐like properties (Azuma et al. 2012; Wang et al. 2017). In non‐small‐cells‐lung‐cancer (NSCLC), NDRG1 is linked to resistance against chemotherapy (e.g., *cis*‐platin) (Du et al. 2018), and targeted therapies against epidermal growth factor receptor (EGFR) (Wang et al. 2017; Wang et al. 2022). Driver mutations in EGFR, frequently found in never‐smoker lung cancer patients, are recognized as promoting factors in NSCLC initiated by particulate matter (PM) exposure (LoPiccolo et al. 2024). These observations point to a critical interplay between NDRG1, EGFR, and PM‐driven tumorigenesis. This connection is of major biomedical relevance, as exposure to air pollution and PM is a global health issue that goes beyond individual control.

Among PM constituents, nickel is of particular concern. Classified as a Class I carcinogen by the International Agency for Research on Cancer (IARC) in 1990 (Begum et al. 2022; Turner et al. 2020), inhaled nickel compounds are associated with elevated risks of lung and nasal cancers (Zambelli et al. 2016). Nickel exposure induces NDRG1 upregulation in lung tissues through activation of the hypoxic response, a hallmark of tumor physiology (Chen et al. 2017; Salnikow et al. 2000; Zambelli and Ciurli 2013). Importantly, NDRG1 has been shown to directly bind Ni(II), providing a mechanistic link between nickel‐induced upregulation and its function as a metal‐binding protein (Beniamino et al. 2022; Zoroddu et al. 2009).

Ni(II) binds specifically to the C‐terminal sequence of NDRG1 (hereafter called NDRG1*C) (Beniamino et al. 2022; Zoroddu et al. 2009), an intrinsically disordered region (IDR) of 84 residues absent in other NDRG orthologs (Beniamino et al. 2022). In contrast, the N‐terminal α/β hydrolase domain is conserved across NDRG family members and structurally characterized through crystallography in NDRG1 (Mustonen et al. 2021), NDRG2 (Hwang et al. 2011), and NDRG3 (Kim et al. 2020). The uniqueness of NDRG1*C suggests that many of the distinctive functions of NDRG1 depend on this region. NDRG1*C contains a 3 × 10‐residues repeat, including three Ni(II)‐binding histidine (His) residues (Beniamino et al. 2022; Mustonen et al. 2021). This region also mediates association with membranes (Mustonen et al. 2021) and contributes to key cancer‐related processes such as invasiveness, adhesion, proliferation, and differentiation (You et al. 2022). The integrity of the NDRG1*C sequence is essential for protein intracellular trafficking, enabling its translocation between the cytoplasm and the nucleus (Kachhap et al. 2007; Park et al. 2018; You et al. 2022). Moreover NDRG1*C contains multiple Ser/Thr phosphorylation sites that add to its regulatory complexity (McCaig et al. 2011; Murray et al. 2004), influencing subcellular localization, protein–protein interactions, and functional outcomes including apoptosis and tumor suppression (You et al. 2022). These observations suggest that NDRG1*C represents a hot spot target for anti‐cancer strategies. However, how phosphorylation of NDRG1*C affects its conformation or molecular interactions with Ni(II) or lipids is still unknown.

The present study provides a detailed biophysical and conformational characterization of the interactions between NDRG1*C and membrane vesicles, and examines how phosphorylation affects its role as a Ni(II)‐ and lipid‐binder module. To achieve these goals, we employed a combination of methods including circular dichroism (CD), nuclear magnetic resonance (NMR), electron paramagnetic resonance (EPR), Fourier‐transform infrared spectroscopy (FTIR), and isothermal titration calorimetry (ITC). By elucidating the molecular effects of phosphorylation and membrane association, this study advances our understanding of how NDRG1*C functions as regulatory switch controlling NDRG1 activity and contribution to cancer progression. Selectively interfering with the phosphorylation‐dependent interactions and conformational states of NDRG1*C could represent a therapeutic strategy to modulate its oncogenic functions.

## 2 RESULTS

Previous studies have shown that NDRG1 is phosphorylated by multiple kinases, such as PKA, PKC, SGK1, CMK‐II, PI3K, and GSK3b (Joshi et al. 2022; Kitowska and Pawełczyk 2010). Phosphorylation has been implicated in regulating diverse aspects of NDRG1 function, such as subcellular localization (Ledet et al. 2021; Park et al. 2018; You et al. 2022), interaction with partners (Guo et al. 2025; Ledet et al. 2021; Weiler et al. 2014), tumor‐promoting activity (Park et al. 2018; Valluri et al. 2023; You et al. 2022), and protein degradation (Guo et al. 2025). However, the molecular mechanisms by which phosphorylation governs these processes remain poorly understood. Notably, phosphorylation sites are clustered within the intrinsically disordered C‐terminal region of NDRG1 (NDRG1*C), with modifications targeting serine and threonine in a manner that is both kinase‐ and cell‐type‐specific.

### 2.1 NDRG1*C is phosphorylated by PKA

**FIGURE 1:**
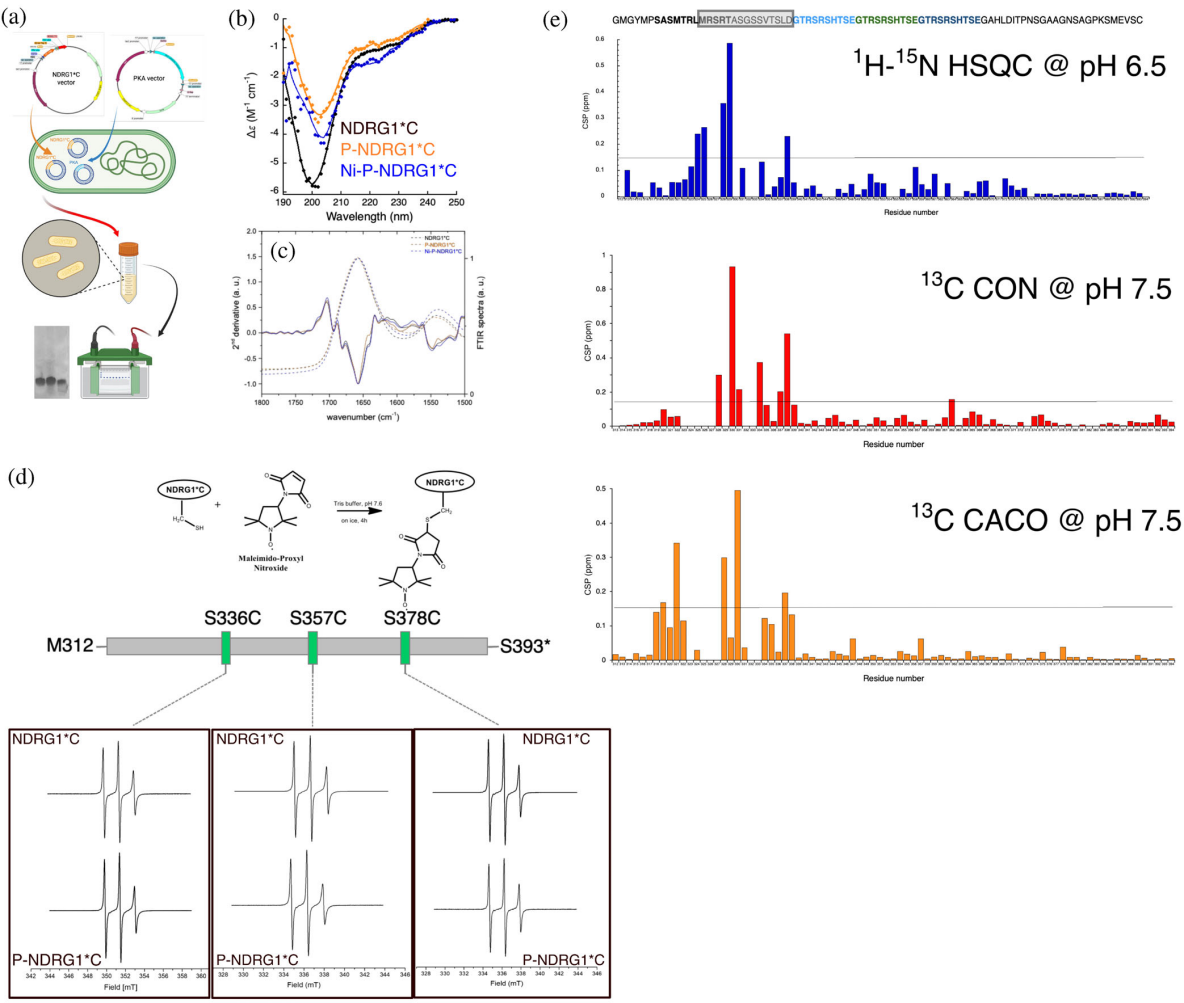
(a) Scheme for in cell NDRG1*C phosphorylation by PKA, whose gene was recombinantly co‐expressed with ndrg1*C in *E. coli*, the phosphorylated protein extracted from bacteria and purified. Created in BioRender. (b) Circular dichroism of NDRG1*C in its unmodified (black), phosphorylated states in the absence (orange), and in the presence (blue) of Ni(II). Filled circles represent experimental points, solid lines are the fits obtained using BestSel. (c) FTIR spectra of NDRG1*C in its unmodified (black) and phosphorylated states in the absence (orange) and in the presence (blue) of Ni(II), dried on ATR crystal. Solid lines represent the experimental data, dashed lines represent the 2nd derivative of the FTIR spectra. (d) Scheme for the site‐directed spin labeling reaction targeting Cys residues with the paramagnetic label Maleimido‐Proxyl (upper panel) and X‐band CW—room temperature EPR spectra of the labeled variants of NDRG1*C in its unmodified and phosphorylated states (lower panels). (e) Chemical shift perturbation analysis of NDRG1*C phosphorylation. Each bar indicates the per residue change in ppm obtained comparing the spectra (^1^H‐^15^N HSQC at pH 6.5, ^13^C CON, and ^13^C CACO at pH 7.5) of the phosphorylated sample with the one of the unmodified protein. The sequence of the protein is reported over the plots: for clarity, the protein sequence containing the phosphorylation sites is enclosed in a box, the sequence with a‐helical propensity is represented in bold, and the repeated sequences are colored in cyan, green, and blue.

To investigate the molecular effects of phosphorylation within NDRG1*C, it is crucial to identify both the post‐translational modification sites and their structural consequences. NDRG1*C is not phosphorylated when it is expressed alone in an *E. coli* system (Beniamino et al. 2022). To obtain a phosphorylated sample, we co‐expressed the protein with the catalytic subunit of human protein kinase A (PKA) in *E. coli*, therefore introducing human kinase activity into the bacterial expression system. The vector expressing the His‐ZZ‐tagged NDRG1*C was co‐transformed into *E. coli* BL21(DE3)‐RIL cells together with a plasmid carrying the gene for the PKA catalytic subunit. Both genes were placed under the control of the T7 promoter, allowing simultaneous induction by IPTG (Figure 1a). Phosphorylated NDRG1*C was successfully purified using a protocol adapted from that of the unmodified protein. The introduction of phosphate groups altered the isoelectric point of the protein, which was reflected in its chromatographic behavior. Specifically, unmodified NDRG1*C could be separated from the affinity tag by cation‐exchange (CIEX) chromatography at pH 7.5, eluting at ca. 200 mM NaCl. In contrast, phosphorylated NDRG1*C did not bind to the negatively charged resin under these conditions. Efficient separation was achieved only at pH 6, where the protein eluted at ca. 100 mM NaCl (Figure S1a, Supporting Information), consistent with a substantial shift in isoelectric point. Mass spectrometry analysis on the purified protein revealed heterogeneity of the phosphorylation pattern, with the sample containing a mixture of NDRG1*C species carrying four (45%) and five (52%) phosphate groups (Figure S1b).

### 2.2 Phosphorylated NDRG1*C maintains its intrinsically disordered behavior

The conformational effects of NDRG1*C phosphorylation were first examined at the level of secondary structure using circular dichroism (CD) and Fourier transform infrared spectroscopy (FTIR). As previously reported, the CD spectrum of NDRG1*C is typical of an intrinsically disordered protein, with secondary structure contents of 5.8% α‐helix, 24.4% β‐strand, and 69.8% disordered (Figure 1b) (Beniamino et al. 2022). Upon phosphorylation, the CD spectrum displayed a slight shift of the ellipticity minimum from 199 to 203 nm, suggesting a gain in secondary structure. Quantitative analysis of the CD spectrum using BestSel confirmed a two‐fold increase in α‐helical content (13.1%), accompanied by a decrease in β‐strand content (17.1%), while the fraction of unordered structure remained essentially constant (69.8%) (Figure 1b). The absence of a pronounced negative minimum at 222 nm, typical for α‐helical content, can be rationalized by considering that, although increasing the α‐helical propensity in relative terms (with the estimated α‐helical content approximately doubling), the protein remains largely disordered, presenting only a minor fraction of the overall conformational ensemble as α‐helix.

The FTIR spectrum of unmodified NDRG1*C showed two major peaks (Figure 1c): the amide I band (ca. 1660 cm^−1^), arising from the C=O stretching, and the amide II band (ca. 1550 cm^−1^), due to N‐H bending. The position of the amide I band is sensitive to secondary structure content (Waeytens et al. 2021) (Table S1). For instance, α‐helices produce a maximum near 1654 cm^−1^, whereas β‐strands yield one close to 1633 cm^−1^. Second‐derivative analysis of the FTIR spectra revealed a decrease in the band at 1635 cm^−1^ upon phosphorylation, consistent with a decrease in the β‐strand content from 33% to 26%. This is consistent with the CD analysis, although FTIR did not clearly detect an increase in α‐helical content. This discrepancy likely reflects the inherent difficulty of distinguishing α‐helix from random coil conformations in FTIR spectra in the absence of deuterium‐exchange experiments (De Meutter and Goormaghtigh 2021).

Protein dynamics were then probed using continuous wave EPR (CW‐EPR) experiments with site‐directed spin labeling (SDSL) (Pierro and Drescher 2023; Torricella et al. 2021). To this end, single‐Cys variants of NDRG1*C were generated by introducing cysteine residues at positions S336, S357, or S378, while the native C394 was removed to avoid non‐specific labeling (Figure 1d). The mutants were labeled with the paramagnetic probe Maleimido‐Proxyl nitroxide producing S336C^Proxyl^, S357C^Proxyl^, or S378C^Proxyl^ variants, enabling site‐specific reporting of local dynamics upstream, within, and downstream of the 3× repeat (3R) sequence (You et al. 2022). CW‐EPR spectra for rapidly rotating nitroxides usually produce three narrow lines of equal width and intensity. As the rotational mobility decreases, line broadening becomes apparent, which is most pronounced for the high‐field line. Therefore, the line shape depends on the rotational mobility of the nitroxide tag that is a sensitive reporter of local structural organization in the vicinity of the labeled residue (Flores Jiménez et al. 2010; Pierro et al. 2024). CW‐EPR spectra of S336C^Proxyl^, S357C^Proxyl^ or S378C^Proxyl^ NDRG1*C variants showed line shapes typical of highly flexible, coil‐like segments (Figure 1d). Simulations using the SimLabel program (Etienne et al. 2023) (Figure S2) yielded a correlation time (*τ*_*c*_) of ca. 0.3 ns for most of the protein population, with a minor fraction of protein molecules showing a more rigid behavior (*τ*_*c*_ = 1.30 ns). Notably, phosphorylation did not significantly alter the dynamic properties of any of the probed sites (Figures 1d and S2).

The effects of phosphorylation were also investigated using NMR chemical shift perturbation (CSP) analysis. ^1^H‐^15^N HSQC spectra of phosphorylated and unmodified NDRG1*C were compared (Beniamino et al. 2022). For the unmodified protein, well‐dispersed spectra could be obtained only at pH 6.5 (Figure S3); at pH 7.5, extensive line broadening suggested exchange phenomena involving backbone amide protons (Beniamino et al. 2022). A similar behavior was obtained for the phosphorylated variant. At pH 6.5, the phosphorylated protein displayed the narrow ^1^H chemical shift dispersion (8.0–8.6 ppm) typical of intrinsically disordered proteins, consistent with the EPR data and indicating that phosphorylation does not induce stable folding; instead, the backbone remains dynamic, undergoing fast exchange among multiple conformations (Figure S3). CSP mapping revealed the strongest perturbations in the N‐terminal region, particularly residues M324‐D338 (Figure 1e). Within this stretch, five residues exhibited CSP >0.15 ppm (M324, R325, T328, A329, D338), while peaks for five others disappeared altogether (S326, R327, S330, S332, S333), likely due to large shifts that prevented their identification in the spectrum. Indeed, five new peaks appeared between 9.1 and 8.8 ppm, consistent with backbone amide shifts induced by intra‐residue hydrogen bonding between amide protons and phosphate groups, a hallmark of phosphorylated residues in extended conformations (Theillet et al. 2012). As reported previously, signals for M312, H345, H355, H365, H371 were absent in the spectrum, due to either conformational exchange (M312) or amide proton exchange (His residues) (Beniamino et al. 2022).

Complementary ^13^C‐detected NMR experiments further confirmed these findings. Comparison of CACO and CON spectra of phosphorylated NDRG1*C with those of the unmodified protein at pH 7.5 (Beniamino et al. 2022) revealed the largest perturbations in the L323–D338 region. In the CON spectrum, six residues showed CSPs >0.15 ppm (T328, S330, G331, V334, L337, D338), while eight disappeared (L323, M324, R325, S326, R327, A329, S332, S333). In the CACO spectrum, five residues were significantly perturbed (S319, T321, T328, S330, L337), and six disappeared (L323, R325, S326, R327, S332, S333) (Figure 1e). This analysis demonstrates that NDRG1*C phosphorylation predominantly occurs within residues M324–D338. This sequence contains five Ser/Thr sites (S326, T328, S330, S332, S333) that undergo major CSPs. Notably, T328 and S330 have previously been identified as phosphorylation sites for multiple kinases (Guo et al. 2025; Ledet et al. 2021; Murray et al. 2004; Park et al. 2018). In contrast, no significant CSPs were detected at S345, S356, and S366 within the 3R sequence, despite their reported phosphorylation in cellular studies (Joshi et al. 2022). Interestingly, the region most affected by phosphorylation overlaps with the ^318^ASMTRLMRS^327^R sequence (Figure 1d), which has a small intrinsic helical propensity (Beniamino et al. 2022). This observation is consistent with the increase in α‐helical content of phosphorylated NDRG1C detected by CD.

To experimentally validate the Ser/Thr residues recognized as phosphorylated by NMR, site‐directed single mutants were produced substituting the five identified Ser/Thr with Ala and determining the number of phosphate groups of each variant by intact‐protein mass spectrometry (Figure S1c). Interestingly, the S326A mutant could not be purified in the phosphorylated form, as the protein was found in inclusion bodies when co‐expressed with PKA. Notably, the mutant was fully soluble when expressed as an unphosphorylated variant, suggesting that, in the presence of a PKA‐specific phosphorylation pattern, the lack of a phosphate group bound to S326 destabilized the protein fold. This observation hints at S326 phosphorylation in the native sequence. It is interesting to note that S326 belongs to the region with α‐helical propensity, as indicated above. In all other cases, the total number of phosphate groups attached to the protein was reduced by one or more units compared to the wild‐type protein, confirming that these residues contribute to the overall phosphorylation pattern (Figure S1c). The S330A variant displayed a marked shift toward species containing only one or two phosphate groups, with a concomitant reduction of the highest‐phosphorylated forms, suggesting that phosphorylation at this position may act as a priming event, facilitating subsequent phosphorylation events.

### 2.3 Phosphorylation changes the Ni(II) binding mode of NDRG1*C

Previously, isothermal titration calorimetry (ITC) studies established that NDRG1*C binds 1–2 Ni(II) ions in a single event with a dissociation constant *K*_*D*_ of ca. 70 μM (Beniamino et al. 2022). The binding was enthalpically driven, suggesting that Ni(II) coordination induces conformational rearrangements in the protein. Nevertheless, CD and NMR analyses showed no evidence of stable secondary structure acquisition within the intrinsically disordered region upon metal binding (Beniamino et al. 2022). NMR data further demonstrated that Ni(II) is coordinated within a paramagnetic site of octahedral or square‐pyramidal geometry involving four histidine residues (H345, H355, H365, H371). This conclusion was supported by the disappearance of NMR signals corresponding to side‐chain and backbone nuclei of these His and neighboring residues (Beniamino et al. 2022).

**FIGURE 2:**
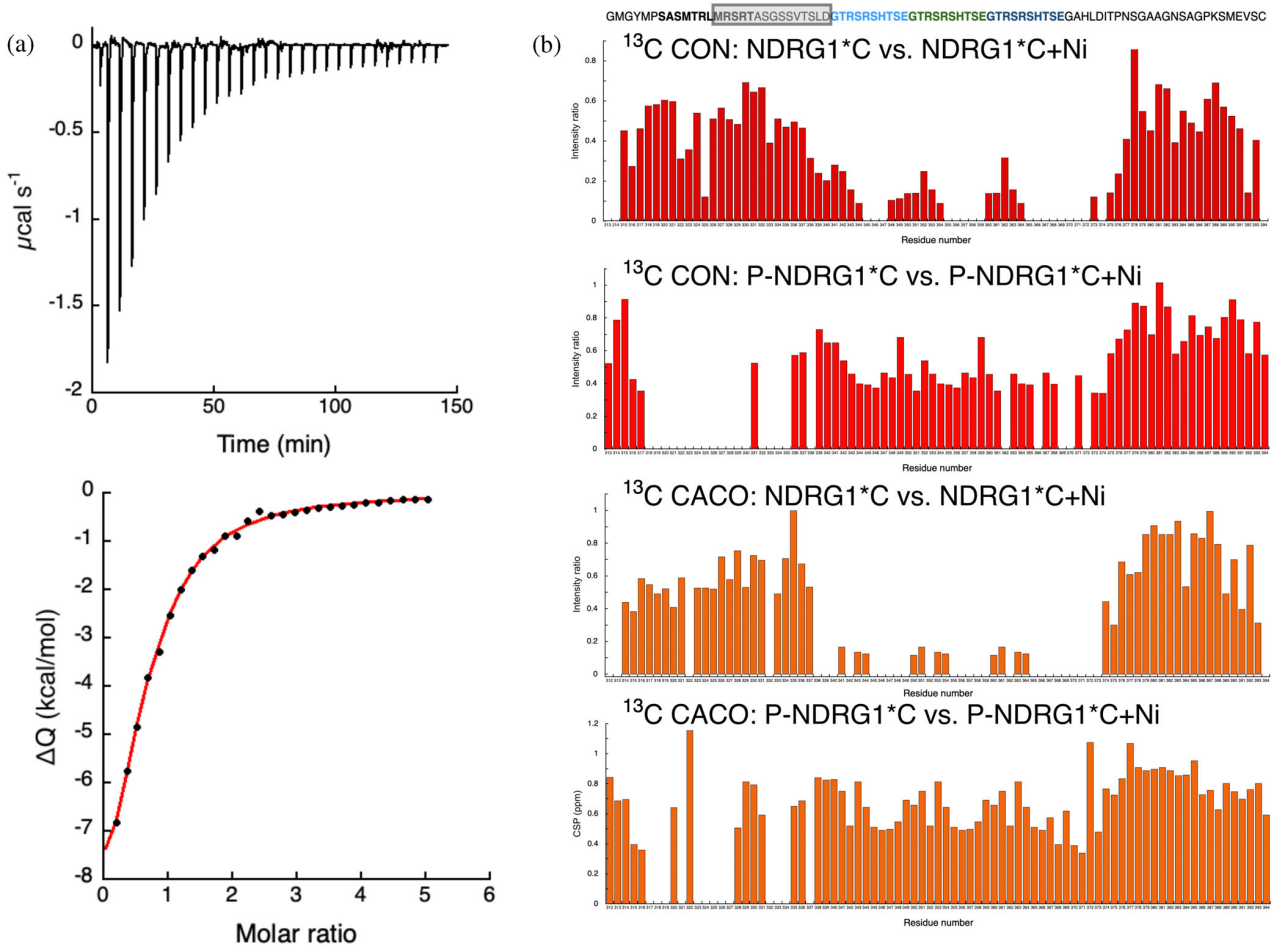
(a) ITC titration data for the binding of Ni(II) to phosphorylated NDRG1*C. Upper panel: raw titration data represent the thermal effect of 10 μL injections of Ni(II) onto or P‐NDRG1*C solution at pH 7.5. Bottom panel: normalized heat of reaction data for the binding events of Ni(II) to P‐NDRG1*C were obtained by integrating the raw data. The solid line represents the best fit of the integrated data, obtained by a nonlinear least‐squares procedure, as described in the text. (b) Intensity ratios of the peaks in the CON spectra and CACO spectra obtained at 700 MHz (16.4 T) by ^13^C direct detection at pH 7.5 for the unmodified and phosphorylated samples, in the absence and presence of one equivalent of Ni(II). The sequence of the protein is reported over the plots: for clarity, the protein sequence containing the phosphorylation sites is enclosed in a box, the sequence with a‐helical propensity is represented in bold, and the repeated sequences are colored in cyan, green, and blue.

To evaluate the effect of phosphorylation on Ni(II) binding, ITC titrations were performed under conditions similar to those previously reported (Beniamino et al. 2022). Control experiments with the unmodified protein reproduced earlier results (Figure S4 and Table S2) (Beniamino et al. 2022). Similarly, titration of Ni(II) into phosphorylated NDRG1*C (Figure 2a) yielded a binding isotherm with a single inflection point consistent with a one‐site binding model. The derived parameters (Table S2) revealed an affinity nearly threefold higher than the unmodified protein (*K*_*D*_ = 13 μM), while thermodynamic values remained similar (Δ*H* = −11.9 kcal mol^−1^; Δ*S* = −17.5 cal mol^−1^ K^−1^), confirming an enthalpy‐driven interaction. Ni(II) binding also altered the structural properties of phosphorylated NDRG1*C. CD spectra showed a shift in ellipticity minimum from 203 to 200 nm, consistent with increased random coil content. BestSel analysis indicated that Ni(II) binding essentially abolished the α‐helical structure (reduced to 1.0%) while increasing β‐sheet content to 31.0% (Figure 1b). FTIR spectroscopy corroborated this finding, with an increased band intensity at 1635 cm^−1^ corresponding to ~38% β‐sheet content (Figure 1c).

At the residue level, Ni(II) binding was analyzed by peak intensity perturbations in ^13^C‐detected NMR spectra (CON and CACO) at pH 7.5. As previously observed for the unmodified protein (Beniamino et al. 2022), Ni(II) addition caused disappearance of several signals without significant chemical shift changes, consistent with binding in a paramagnetic site that does not alter backbone conformation. In the unmodified protein, the affected residues clustered around the four His residues within the central decapeptide repeats, with side chains of D338, H345, E348, H355, D358, H365, D368, H371, D373, N377, and C394 implicated in metal coordination (Figure 2b) (Beniamino et al. 2022). In sharp contrast, Ni(II) binding to phosphorylated NDRG1*C shifted the perturbation pattern. Resonances from residues S318–T335 disappeared upon metal binding, whereas the histidine‐rich decapeptide region was minimally affected. Only signals from S362, T366, G369, A370, and L372 were lost in the CON spectrum (but not CACO), suggesting minor contributions from this region (Figure 2b). These results indicate that phosphorylation completely remodels the Ni(II)‐binding mode of NDRG1*C, diverting the primary binding site from the histidine cluster to the phosphorylated N‐terminal region. This likely arises from electrostatic stabilization of Ni(II) by the negatively charged phosphate groups.

### 2.4 Phosphorylation abolishes NDRG1*C interaction with liposomes

**FIGURE 3:**
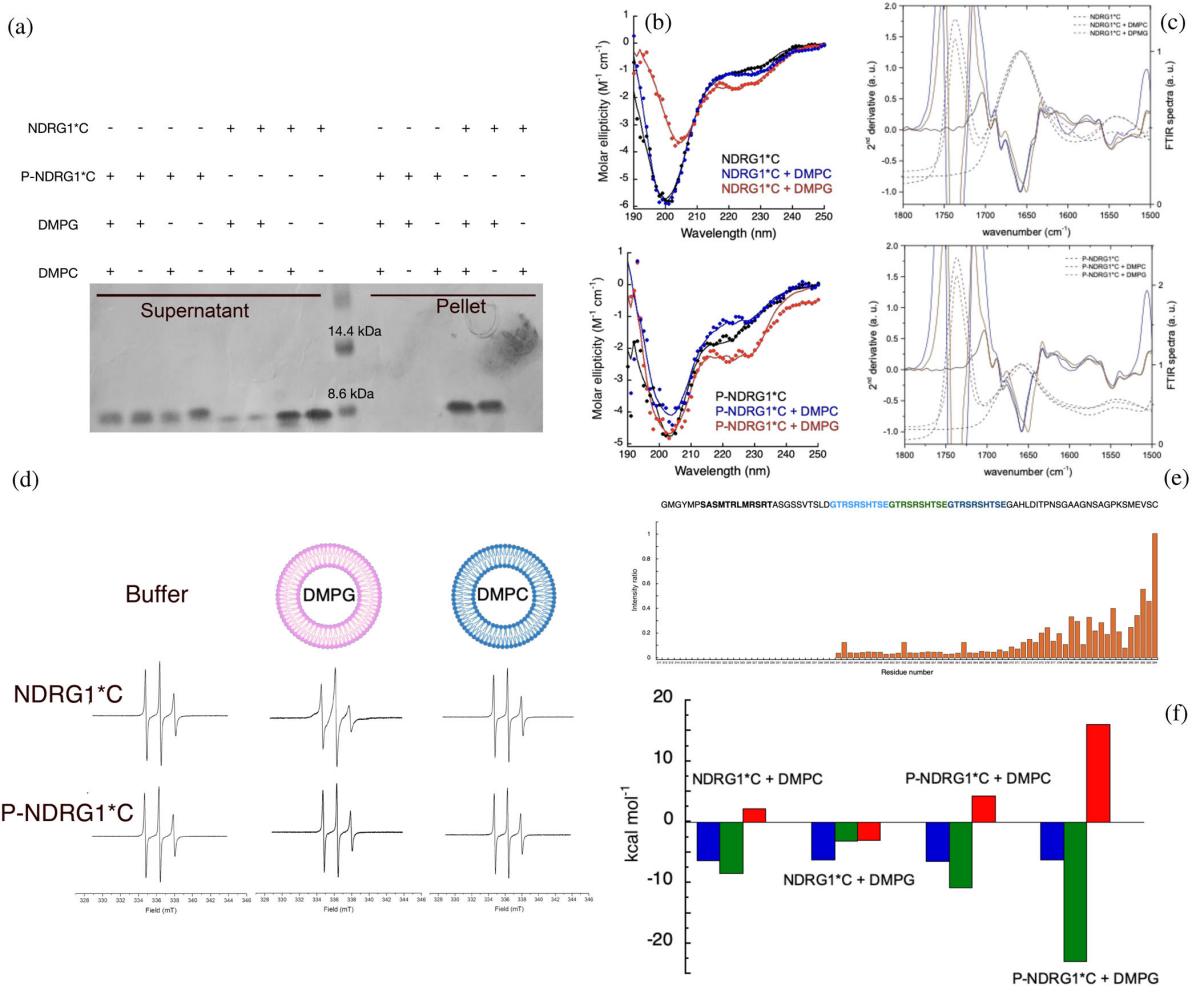
(a) Co‐sedimentation analysis of unmodified and phosphorylated NDRG1*C and liposomes with different lipid composition. (b) Circular dichroism of NDRG1*C (upper panel) and P‐NDRG1*C (bottom panel) in solution (black), and in the presence of DMPC (blue) and DMPG (red)‐based liposomes. Filled circles represent experimental points, solid lines are the fits obtained using BestSel. (c) FTIR spectra of NDRG1*C (upper panel) and P‐NDRG1*C (bottom panel) dried on ATR crystal (black), and in the presence of DMPC (blue) and DMPG (orange)‐based liposomes. Solid lines represent the experimental data, dashed lines represent the 2nd derivative of the FTIR spectra. (d) CW‐EPR spectra of the S336C^Proxyl^ NDRG1*C in its unmodified and phosphorylated states and in the absence and in the presence of DMPC and DMPG‐based liposomes. (e) Intensity ratios of the peaks in the CACO spectra obtained at 700 MHz (16.4 T) by ^13^C direct detection at pH 7.5 for NDRG1*C, in the absence and presence of DMPG‐based liposomes. The sequence of the protein is reported over the plots: for clarity, the protein sequence containing the phosphorylation sites is enclosed in a box, the sequence with α‐helical propensity is represented in bold, and the repeated sequences are colored in cyan, green, and blue. (f) Thermodynamic parameters (Δ*G*, blue; Δ*H*, green; −*T*Δ*S*, red) obtained from the fit of the binding isotherms obtained by ITC represented in Figure S10 and reported in detail in Table S2.

Previous studies on full‐length NDRG1 showed that the protein interacts with negatively charged vesicles composed of a 1:1 mixture of the neutral lipid 1,2‐dimyristoyl‐sn‐glycero‐3‐phosphocholine (DMPC) and the anionic lipid 1,2‐dimyristoyl‐sn‐glycero‐3‐phosphoglycerol (DMPG). Under these conditions, approximately half of the protein co‐precipitated with the vesicles (Mustonen et al. 2021). In contrast, vesicles with reduced negative charge (DMPC:DMPG 9:1) pulled down only a small fraction of NDRG1, indicating that electrostatics drive protein‐lipid interactions (Mustonen et al. 2021). Importantly, constructs lacking the C‐terminal region (from residue 319 onward) were unable to interact with vesicles regardless of their charge (Mustonen et al. 2021), highlighting the critical role of the C‐terminal tail. These findings prompted us to investigate the molecular details of the interaction of NDRG1*C with liposomes and to evaluate how phosphorylation modulates this process. When incubated with negatively charged vesicles (DMPG alone or DMPC:DMPG, 1:1), unmodified NDRG1*C was recovered almost entirely in the pellet after centrifugation (Figure 3a). In contrast, neutral DMPC vesicles failed to co‐precipitate the protein, confirming the requirement for lipid negative charge. Strikingly, phosphorylation completely abolished vesicle binding, irrespective of lipid composition (Figure 3a), suggesting that the negatively charged phosphate groups introduced in the N‐terminal region repel anionic membranes and disrupt lipid association. Addition of Ni(II) does not produce any difference in this pattern, suggesting that Ni(II) does not change the ability of NDRG1*C to interact with membranes, nor the effects of protein phosphorylation on abrogating micelle binding (Figure S5). Spectroscopic analyses revealed conformational changes associated with vesicle binding. CD measurements showed that interaction with DMPG‐containing vesicles increased the α‐helical content of NDRG1*C from 6% to 15% (Figure 3b). FTIR spectra corroborated this observation, with a shift of the amide I band from 1660 to 1650 cm^−1^, even though quantitative analysis did not detect an increase in the α‐helical content, due to the difficulty in distinguishing between α‐helix and random coil structures, as discussed above (Figure 3c). By contrast, neutral DMPC vesicles, or phosphorylated protein in the presence of either DMPC or DMPG vesicles, produced only minor structural effects (Figure 3b,c). These results align with earlier findings for full‐length NDRG1 (Mustonen et al. 2021) and demonstrate that conformational rearrangements upon lipid binding localize to the C‐terminal sequence. As observed in the absence of lipids, Ni(II)‐addition has a modest effect on the spectra that decreased the a‐helical content from 15% to 12% (NDRG1*C) and from 12% to 10% (phosphorylated NDRG1*C) for DMPG‐based vesicles and from 5.4% to 4.2% (NDRG1*C) and from 7.4% to 7.1% (phosphorylated NDRG1*C) for DMPC‐based vesicles (Figure S6a,b). FTIR spectroscopy corroborated this finding (Figure S6c,d): very slight changes in the FTIR spectra collected in the presence of both lipids and Ni(II) are visible, with a shift of the band below 1650 cm^−1^, indicating a minor decrease of the α‐helix and increase in β‐sheet contents. These modifications are more important with DMPG than with DMPC. Dynamic changes were further assessed by CW‐EPR spectroscopy of nitroxide‐labeled NDRG1*C variants. Spin labels at positions 357 and 378 showed little change in the presence of DMPG vesicles (Figure S7). In contrast, S336C^Proxyl^ variant exhibited significant spectral broadening (Figure 3d), reflecting reduced probe mobility and conformational rearrangement in this region. Spectral simulations (Figure S2) revealed that most of the protein population adopted a slower dynamic regime (*τ*_*c*_ = 2.51 ns), while a minor fraction remained in fast exchange (*τ*_*c*_ = 0.19 ns), consistent with vesicle‐induced stabilization of a more rigid conformation. These two components most likely represent distinct protein conformational sub‐states rather than rotamers: a *fast*, highly disordered state (*τ*_*c*_ = 0.19 ns) and a *slow*, more rigid state (*τ*_*c*_ = 2.51 ns), which predominates. This interpretation is further supported by the S336C^Proxyl^ variant, in which glycerol addition increased the proportion of the slow component (*τ*_*c*_ = 1.3 ns, see above) from 47% to 71%. This shift is consistent with osmolyte‐induced redistribution of the conformational landscape toward a more compact state, but not with increased structural rigidity, which would manifest as a change in correlation time (Flores Jiménez et al. 2010; Pierro et al. 2023) (Figure S8). Together, these findings suggest that the region surrounding S336 undergoes induced folding upon interaction with DMPG vesicles. No such effect was observed with neutral DMPC vesicles or phosphorylated variants (Figures 3d and S2), indicating that lipid interactions specifically involve the negatively charged vesicles with the unphosphorylated N‐terminal segment with intrinsic α‐helical propensity.

Residue‐level mapping by ^13^C‐detected NMR further confirmed these findings. In the presence of DMPG‐containing vesicles, signals corresponding to residues 311–340 disappeared from CACO spectra, consistent with binding to large, slow‐tumbling vesicles (Figure 3e). Signal intensities progressively recovered beyond residue 341, indicating greater mobility in the C‐terminal half. In contrast, DMPC vesicles produced only modest, non‐systematic perturbations, with no peak disappearance (Figure S9), supporting the lack of specific interaction.

Finally, the effect of lipid binding on Ni(II) coordination was examined by ITC. The affinity of NDRG1*C for Ni(II) remained ~20 μM in the presence of either DMPC or DMPG vesicles (Figure S10 and Table S2). However, the thermodynamic profile changed in the presence of DMPG: the entropy contribution (Δ*S*) switched from negative to positive (Figure 3f), suggesting that vesicle binding prevents Ni(II)‐induced conformational rearrangements.

## 3 DISCUSSION AND CONCLUSIONS

The structural plasticity of NDRG1*C, the intrinsically disordered C‐terminal region of NDRG1, makes it a key regulatory hub and a hot spot for therapeutic targeting in drug screening campaigns. Understanding how its conformational ensemble responds to environmental changes, molecular interactions, and post‐translational modifications is critical for drug development. NDRG1*C is phosphorylated by multiple kinases, including PKA, PKC (Agarwala et al. 2000), SGK1, GSK3β (Murray et al. 2004), and PIM1 (Ledet et al. 2021). As demonstrated for other intrinsically disordered regions (IDRs) (Semenyuk 2021), phosphorylation alters charge density, electrostatics, and dynamics, essentially tuning protein functions. The dual role of NDRG1 as either tumor suppressor or oncogene can be explained by the kinase‐specific phosphorylation patterns that vary across tissue and cellular types.

This study explores how phosphorylation affects the conformational ensemble of NDRG1*C, its interaction with Ni(II) ions, and its association with membranes. Previous studies at the cellular level have shown that multisite phosphorylation regulates NDRG1 localization, interactions, and cancer‐related functions (Joshi et al. 2022; Kitowska and Pawełczyk 2010), but the structural and molecular consequences of these modifications remain fairly unexplored. The present work moves from the previously reported cellular level approaches, into a molecular level view of the protein, using a combined approach of biophysical and spectroscopic methods to dissect the effects of phosphorylation on NDRG1*C.

**FIGURE 4:**
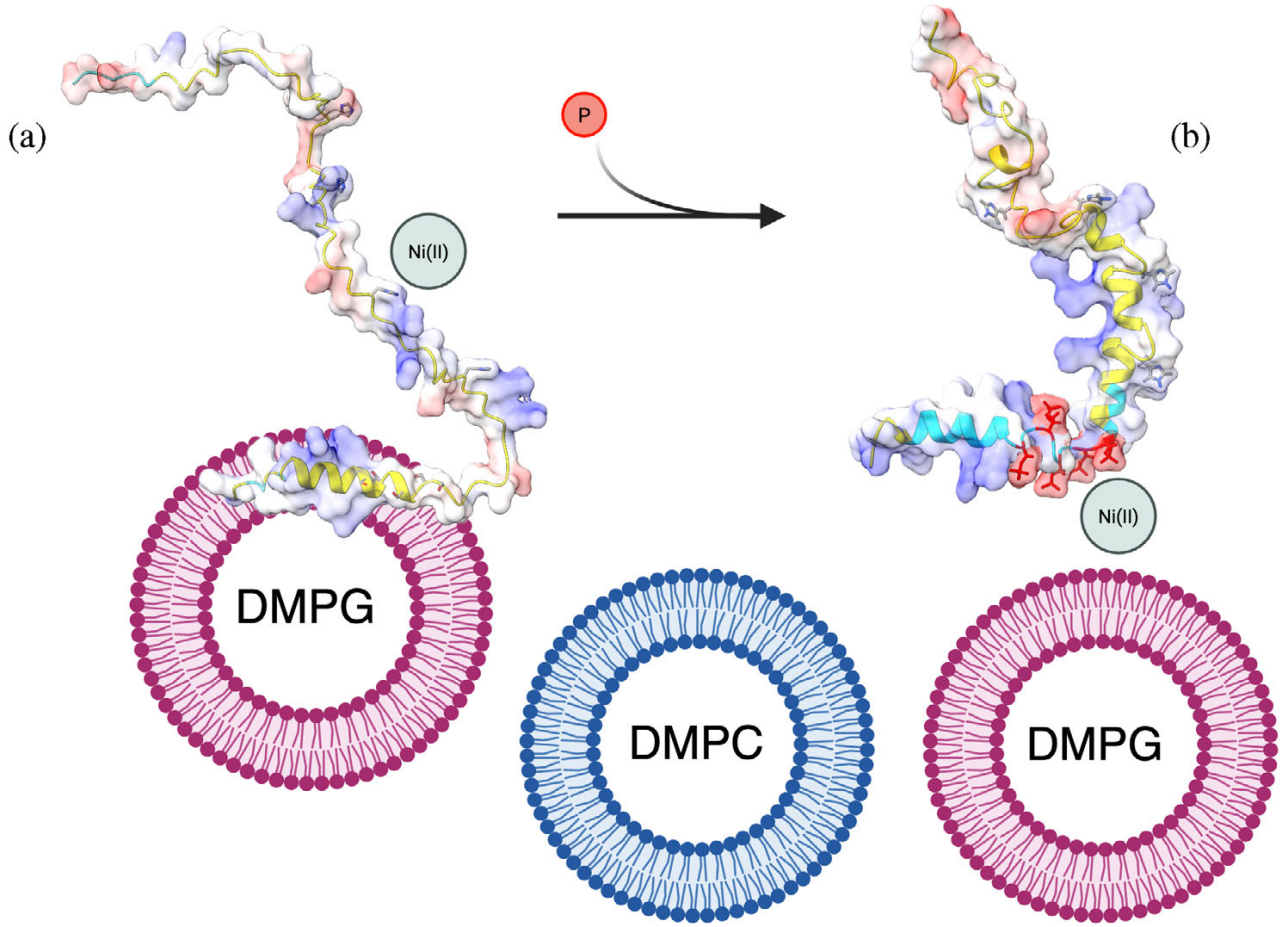
Schematic representation of the effects of phosphorylation on structural transition, vesicle interaction and Ni(II) binding by NDRG1*C. Alpha fold prediction of the unmodified NDRG1*C (a) and of the phosphorylated NDRG1*C (b). Ribbons are colored according to the pLDDT score (yellow = 70 > plDDT > 50; cyan = 90 > plDDT > 70), while surface is colored according to electrostatic potential. Ni(II) binding His residues, Ser and Thr residues that are phosphorylated according to NMR results, or phosphate groups, are represented. Created in BioRender.

The phosphorylated protein was obtained by recombinant co‐expression of NDRG1*C with PKA in *E. coli*. The purified protein contains four or five phosphate moieties, localized upstream of the 3R peptide from M324 to D338 (Figure 4a), in a sequence that includes five Ser/Thr residues (S326, T328, S330, S332, S333). The identified poly‐phosphorylated region partially superimposes to the sequence displaying α‐helical propensity (Figure 4a) (Beniamino et al. 2022). Phosphorylation doubles the α‐helical content of the protein, suggesting that the addition of multiple negative charges modulates the α‐helical formation, without a general induction of protein folding. Consistently, AlphaFold (https://alphafoldserver.com/) predictions suggested an increase in local α‐helical content near the phosphorylated region, matching CD quantification: while for the unmodified NDRG1*C a short α‐helix is predicted with low confidence (pLDDT <70) between residues 8 and 19 (11% of the sequence vs. CD quantification of 6%; Figure 4a), phosphorylation extends the α‐helical estimate to almost half of the protein sequence (Figure 4b), and increases the confidence of the helical prediction for 14 residues (pLDDT >70), which represent 17% of the protein structure, similar to CD quantification of α‐helical content (13%). The agreement between the phosphorylation‐induced increase and localization of the helical content by AlphaFold analysis and by the experimental observation supports the complementary value of computational prediction.

Phosphorylation of T328 and S330 has been previously linked to altered protein localization or cancer aggressiveness (Ledet et al. 2021; Park et al. 2018). Other studies found that SGK1 additionally phosphorylates S336, as well as T346, T356, and T366 in the 3R sequence (Martinez‐Lopez et al. 2023; Murray et al. 2004). These latter modifications prime the protein for further phosphorylation by GSK3β at S342, S352, and S362, also found in the 3R sequence (Murray et al. 2004). Here, NMR and site‐directed mutagenesis coupled to MS indicate that only the Ser/Thr residues clustered near the N‐terminus were phosphorylated, while no significant modification was detected in the 3R region. This discrepancy could reflect differences in the kinase specificity between PKA and SGK1, or it might arise from the distinct methodological approaches used to investigate protein phosphorylation. Previous studies, which primarily relied on radioactive phosphate incorporation, mass spectrometry of proteolytic fragments, or antibody‐based detection of phosphorylated residues, can identify even lowly populated phosphorylation states. In contrast, NMR‐based analyses are more sensitive to the predominant molecular species, and therefore may not capture minor phosphorylated forms.

The structural and regulatory consequences of NDRG1*C phosphorylation are complex and remain unexplored. In prostate, pancreatic, colon, hepatocellular and breast carcinoma cells, phosphorylation of S330 has been associated either with increased nuclear localization (Park et al. 2018; Sevinsky et al. 2018). A subsequent study found that, differently, the rate of S330 phosphorylation is inversely proportional to the nuclear localization of NDRG1 in prostate cancer cells, and directly proportional to the protein sensitivity to proteolytic degradation (Ledet et al. 2021). In head and neck cancer, mutation of S342, S352, and S362 decreases the cell motility and invasion, consistent with the oncogenic activity of phosphorylation (You et al. 2022). NDRG1*C phosphorylation in multiple protein sites is induced under hypoxic conditions in renal cell carcinoma. Phosphorylation of T328/S330 and T346/T356/T366, but not S342/S352/S362, causes an increase of invasiveness and metastatic phenotypes in renal cells, impairing the tumor suppressive role of NDRG1, and underscoring the critical role of NDRG1*C phosphorylation status in determining tumor behavior (Guo et al. 2025). These findings emphasize the context‐dependent outcomes of NDRG1*C modification.

NDRG1 function is also tightly linked to nickel‐driven oncogenesis, especially in non‐small‐cell lung cancer (NSCLC) cells. Not only nickel increases NDRG1 expression through the hypoxia pathway governed by HIF1α (Saponaro et al. 2025), but it also promotes NDRG1 phosphorylation through increasing the level of phosphatidyl inositol‐3‐phosphate (PI3P), which in turn activates SGK1 activity (Kitowska and Pawełczyk 2010). In addition, Ni(II) ions bind one His residue in each of the 3R repeats of NDRG1*C and an additional histidine found downstream the 3R sequence (Beniamino et al. 2022). Here, we show that phosphorylation profoundly alters this Ni(II) binding mode: while affinity moderately increases, Ni(II) relocates to the phosphorylated N‐terminal segment of NDRG1*C, likely driven by electrostatic attraction to the negatively charged phosphate groups. Intriguingly, Ni(II) binding reverses the α‐helical stabilization induced by phosphorylation, decreasing the helical content of the phosphorylated protein. These effects may be physiologically relevant for the cellular response to Ni(II) exposure in NSCLC.

Another critical function of NDRG1 involves membrane and lipid interaction. In breast cancer cells, NDRG1 controls lipid droplets accumulation contributing to cancer aggressiveness (Sevinsky et al. 2018). NDRG1 also regulates cholesterol uptake by modulating endosomal trafficking that determines the presence of low‐density lipoprotein (LDR) receptor in the plasma membrane (Pietiäinen et al. 2013). In prostate and breast cancer models, NDRG1 co‐localizes with the membrane‐bound Rab4a GTPase in endosomes, through direct interaction with lipids, especially with anionic phosphatidyl‐inositol‐4‐phosphate (PI4P) (Kachhap et al. 2007). In addition, NDRG1 expression is induced during adipocyte differentiation, which is promoted by protein phosphorylation (Cai et al. 2017), indicating a link between NDRG1*C phosphorylation and lipid metabolism. Considering that altered lipid metabolism is associated with aggressive cancer phenotypes and poor prognoses (Eltayeb et al. 2022; Merino Salvador et al. 2017), NDRG1*C phosphorylation may directly link the oncogenic role of NDRG1 to lung cancer and lipid regulation.

Structural and biophysical analyses have evidenced that NDRG1 binds artificial membranes with a preference for negatively charged vesicles, and that deletion of NDRG1*C abrogates such interaction, suggesting that the C‐terminal sequence is directly involved in membrane binding (Mustonen et al. 2021). The present study demonstrates that NDRG1*C co‐precipitates with liposomes produced with anionic lipids (DMPG) or containing 50% of anionic lipids (DMPC:DMPG), while it does not bind neutral lipids‐based vesicles (DMPC). Protein‐lipids interaction is completely abolished when the protein is phosphorylated, consistently with electrostatic repulsion from the multiple phosphate groups bound to the five Ser/Thr residues near the N‐terminal of NDRG1*C. This suggests that protein‐lipid interaction involves the phosphorylated sequence, and that phosphorylation may act as an electrostatic switch that detaches NDRG1*C from the membrane. NMR intensity perturbations revealed that residues within the phosphorylated N‐terminal half of NDRG1*C associate with vesicles, while the C‐terminal half remains flexible. CW‐EPR further confirmed decreased mobility of the probe attached near the phosphorylation sites upon DMPG binding, supporting induced folding in this region. The sequence binding the lipids also contains the stretch that presents a α‐helical propensity, and lipid association further increases the α‐helical content of NDRG1*C. Notably, vesicle binding also alters the thermodynamic profile of Ni(II) interaction, switching entropy from negative to positive, although leaving the metal binding affinity substantially invariant, suggesting that the effect of Ni(II) on NDRG1*C can be related to a conformational change distinct for the free polypeptide or the membrane‐bound protein. Combined with the fact that phosphorylation prevents membrane binding and reroutes Ni(II) binding toward the membrane interacting region, these data suggest that lipid engagement counteracts Ni(II)‐induced conformational changes.

It is noteworthy that domains rich in negatively charged lipids are often present and functionally relevant in physiological membranes. An example is lipid rafts, dynamic membrane domains enriched in cholesterol and negatively charged glycosphingolipids, which play roles in signaling and vesicle biogenesis, including the formation of exosomes (Anselmo et al. 2025; De Gassart et al. 2003). Similarly, extracellular vesicles such as exosomes frequently contain high levels of phosphatidylserine and other anionic phospholipids, which influence membrane interactions and cellular uptake (Skotland et al. 2019). Moreover, the asymmetric distribution of negatively charged lipids such as phosphatidylserine and phosphoinositides is known to contribute to directing the localization of proteins with basic regions to specific membrane compartments, especially for IDRs (Ma et al. 2017; Yeung et al. 2008). Therefore, although the artificial membranes used in this work are not physiological by themselves, the discovery that NDRG1*C preferentially binds to negatively charged membranes with electrostatic interactions is relevant for understanding its biological behaviors, especially in contexts where negatively charged lipids are locally concentrated.

Altogether, our findings supports a model in which NDRG1 vesicle binding, and consequently protein localization, lipid trafficking and storage, and Ni(II) interactions are regulated by phosphorylation. Using a multimodal biophysical approach, we propose that NDRG1*C serves as a functional node that links membrane recognition, metabolic reprogramming, and nickel responsiveness to tumor aggressiveness. The increased proteasomal degradation of phosphorylated NDRG1 (Guo et al. 2025; Ledet et al. 2021) may reflect its loss of membrane association, rendering the soluble form more accessible to proteolytic pathways. It is known that phosphorylation close to nuclear localization signals (NLSs) of a nuclear protein might influence its recognition and binding by importin‐a/importin‐b translocation system, regulating the protein subcellular localization (Stewart 2007). In silico analysis, performed using cNLS Mapper (Kosugi et al. 2009) or NLStradamus (Nguyen Ba et al. 2009), did not identify any canonical NLS within NDRG1, consistent with a non‐canonical or regulated nuclear import mechanism. Several Ser/Thr residues, including T328 and S330, scored positively in 14‐3‐3 binding predictions using 14‐3‐3‐Pred (Madeira et al. 2015). As 14‐3‐3 proteins are signaling molecules that regulate a huge number of cellular functions, often by interacting with phosphorylated proteins (Yaffe 2002), including nuclear localization (Faul et al. 2005; Sekimoto et al. 2004). Therefore, this analysis suggests that phosphorylation may regulate NDRG1 localization indirectly through phospho‐dependent adaptor interactions. A more recent work found that NDRG1 can facilitate the nuclear import of binding partners through protein–protein interactions even without any canonical NLS: interaction of NDRG1 C‐terminal sequence with the chaperone HSC70 promoted nuclear import of viral proteins. This observation suggests that NDRG1 may participate in regulated nuclear trafficking through multiprotein complexes rather than intrinsic targeting motifs (Zhang et al. 2023a). In endothelial and in hepatocellular carcinoma cells NDRG1 interacts with the orphan nuclear receptor Nur77, functionally inhibiting its transcriptional activity, highlighting that it can engage in functionally significant protein–protein interactions with nuclear factors (Lu et al. 2015; Zhang et al. 2023b). Finally, preliminary data reported an association between NDRG1 and importin‐α/β1 in neuronal cells by co‐immunoprecipitation experiments, supporting the idea that NDRG1 can engage with components of the nuclear transport machinery, potentially through indirect or context‐dependent mechanisms involving protein interactions (Lee et al. 2020).

In conclusion, NDRG1*C emerges as a dynamic regulatory module that integrates multisite phosphorylation, Ni(II) coordination, and lipid interaction to dictate NDRG1 localization and function. Modeling these mechanisms provides new opportunities for therapeutic strategies in hypoxia‐driven and lipid‐rewired cancers.

## 4 METHODS

### 4.1 Construction of the pCDF_Duet‐PKA Cat vector

The cDNA encoding the catalytic subunit of murine PKA (UniProt ID: P05132) was purchased from Eurofins and inserted into the pCDF_Duet vector between BamHI and HindIII restriction sites to make the *pCDF_Duet‐pka* expression vector.

### 4.2 Phosphorylated NDRG1*C expression and purification

Large‐scale expression of the C‐terminal peptide NDRG1*C (residues 312–394) was obtained as previously reported (Beniamino et al. 2022). The expression of the phosphorylated protein was obtained by sequential transformation of *E. coli* BL21(DE3)RIL competent cells with the pETZZ‐ndrg1*C and *pCDF_Duet‐pka* vectors. Double transformed bacteria were cultured in 2 L of lysogeny broth (LB) at 37°C, supplemented with 30 μg/mL kanamycin, 34 μg/mL chloramphenicol, and 100 μg/mL streptomycin. To enhance protein yield, the preculture medium was enriched with 1% glucose. Protein expression, cell lysis, affinity chromatography, buffer exchange and TEV protease cleavage to remove the tag were carried out under the same conditions as for the unmodified protein (Beniamino et al. 2022), with the only difference of the final pH, which was kept at 6.0. Separation of the protein from the fusion tag was achieved via cation exchange chromatography at pH 6.0. The protein was eluted with a linear NaCl gradient ranging from 0 to 1M. Fractions containing the phosphorylated protein were concentrated using a 3 kDa molecular weight cutoff (MWCO) Centricon ultrafiltration device, followed by further purification using a Superdex 75 XK 10/300 column (GE Healthcare) equilibrated with the working buffer (20 mM HEPES pH 7.5, 150 mM NaCl, 1 mM TCEP). Mass spectrometry was used to verify protein phosphorylation and indicated that the protein sample contains four or five phosphate groups in equimolar amounts. Protein concentration was determined by measuring the light absorbance at 280 nm using an extinction coefficient of 2450 M^−1^ cm^−1^, as previously determined (Beniamino et al. 2022). Protein purity was confirmed by SDS‐PAGE analysis. The final yield of the purified protein was approximately 3 mg/L of initial culture.

### 4.3 Lipid preparation

DMPG (1,2‐dimyristoyl‐sn‐glycero‐3‐phospho‐(1′‐rac‐glycerol), sodium salt) and DMPC (1,2‐dimyristoyl‐sn‐glycero‐3‐phosphocholine) were purchased from Avanti Polar Lipids. Lipid stock solutions were prepared by dissolving dry lipids in organic solvents: DMPG was dissolved in a chloroform and methanol mixture (6:1 v/v), while DMPC was dissolved in pure chloroform. Lipid mixtures were then prepared at the desired molar ratios from these solutions and dried under a stream of nitrogen. The dried lipid films were rehydrated in a buffer containing 20 mM HEPES, 150 mM NaCl (or TrisHCl for EPR measurements), at pH 7.5 at concentrations suitable for subsequent analyses. This rehydration process initially produced multilamellar vesicles (MLVs). To improve the homogeneity of the suspension, which presented vesicles with different dimensions as verified with dynamic light scattering, the samples were subjected to five cycles of freezing at −80°C and thawing at 37°C, promoting the formation of large unilamellar vesicles (LUVs). For further refinement, the vesicles were extruded at 60–65°C using an extruder (Avanti Polar Lipids) equipped with 100 nm pore‐sized membranes, resulting in a final solution consisting of small unilamellar vesicles (SUVs), which were used in the subsequent experiments.

### 4.4 Lipid Co‐sedimentation assay

Lipid vesicles (1 mM) were mixed with 10 μM protein in 20 mM HEPES pH 7.5, 150 mM NaCl, 1 mM TCEP. The samples were incubated for 1 h at room temperature, followed by lipid precipitation using ultracentrifugation at 434,500*g* for 1 h at 15°C using an Optima 130 K ultracentrifuge (Beckman Coulter) equipped with an MLA‐130 fixed‐angle rotor. Aliquots from the supernatants and from the pellets resuspended in the protein buffer were analyzed by SDS‐PAGE.

### 4.5 Circular Dichroism spectroscopy

The secondary structure of the protein in its phosphorylated state, as well as its structure in both native and phosphorylated forms in the presence of lipids (protein: lipids ratio 1:100) and NiSO_4_ was analyzed using the JASCO J810 spectropolarimeter, with a scan speed of 100 nm/min, response time of 1 s, band width of 10 nm, data pitch every 0.2 nm. For each experiment, ten spectra were accumulated and averaged to increase the s/n ratio. All protein solutions (110 μM) were prepared in a buffer containing 20 mM HEPES pH 7.5, 150 mM NaCl, and 1 mM TCEP. Circular dichroism (CD) spectra were recorded using a cuvette with a 0.1 mm optical path length at 25°C, over a wavelength range of 260–190 nm, with data points collected at 0.2 nm intervals. The BestSel (Best Structure Selection) software was employed to quantify the structural composition of the protein under the different conditions.

### 4.6 Isothermal titration calorimetry

The interaction between Ni(II) and unmodified or phosphorylated NDRG1*C was investigated at 25°C using a high‐sensitivity VP‐ITC microcalorimeter (MicroCal) equipped with a 310 μL computer‐controlled microsyringe. Protein solutions (30–40 μM concentration diluted in 20 mM HEPES pH 7.5, 150 mM TCEP) were introduced into the sample cell (1.4093 mL), and a series of 29 × 10 μL aliquots of 800 μM NiSO_4_, diluted in the protein buffer, were injected. The same experiments were performed in the presence of DMPC or DMPG SUVs (protein: lipid ratio 1:100). Each injection was followed by a 300 s interval, allowing the system to reach thermal equilibrium. Data were analyzed using the Origin software package (MicroCal), with curve fitting performed via a nonlinear least squares minimization algorithm to fit theoretical binding models. The *χ* ^2^ parameter was used to assess the quality of the fit. Δ*H*, *K*_*A*_, and *n* were the fitting parameters. The entropy change (Δ*S*) and Gibbs free energy change (Δ*G*) were derived using the equations Δ*G* = −*RT*ln*K*_*A*_ (*R* = 1.9872 cal mol^−1^ K^−1^, *T* = 298 K) and Δ*G* = Δ*H* − *T*Δ*S*. The values obtained for ∆*H* and ∆*S* are apparent, and include contributions not only from metal binding, but also from associated events such as protonation/deprotonation of the amino acid residues involved in the binding and consequent change in the buffer ionization state.

### 4.7 Fourier transform infrared spectroscopy

Fourier transform infrared spectroscopy (FTIR) measurements were acquired on Tensor27 spectrophotometer (Bruker) equipped with a LN2‐MCT detector and a single reflection diamond ATR golden‐gate (Specac). The samples were prepared in 5 mM HEPES buffer at pH 7.5, 2 μL of sample were deposited on the diamond, dried with nitrogen flow and spectra were recorded with 64 scans. The lipids were prepared in the same buffer as the sample at a concentration of 15 mM. They were added at a protein: lipid molar ratio of 1:100. A water vapor reference spectrum was subtracted to remove water vapor, and the spectra are scaled at 1656 cm^−1^. The 2nd derivative with a smoothing of 8 cm^−1^ was done using Kinetics (a software developed in our lab working in MatLab).

### 4.8 Site‐directed spin labeling

Site‐directed mutagenesis was used to introduce cysteine residues in specific positions of the protein to map structural dynamic features of different regions of NDRG1*C by EPR spectroscopy. Considering that NDRG1*C contains only one natural occurring cysteine (Cys 394, the last residue), we produced three variants, each carrying a single Cys residue available for labeling, in which Cys394 was mutated to an Ala residue: Ser336Cys/Cys394Ala, Ser357Cys/Cys394Ala, and Ser378Cys/Cys394Ala. Prior to spin labeling, 100 nmol of protein were treated with an excess of TCEP (20×) at 4°C for 30 min to reduce the cysteine moieties, then removed by PD10 desalting column (GE Healthcare), against freshly prepared Tris–HCl buffer (20 mM pH = 7.5, containing 150 mM NaCl). The fractions containing the protein were pooled and incubated with Maleimido‐Proxyl nitroxide (Sigma‐Aldrich; Figure 1d) at 10‐fold molar excess with respect to the protein concentration. The reaction mixture was gently stirred for at least 4 h at 4°C. A second PD10 desalting column was used as described above to remove the excess of unbound spin label. The collected fractions were checked by EPR spectroscopy and polished by centrifugation in 2 mL Vivaspin concentrators 3 kDa MWCO (Sartorius) 6000*g* for 15 min. The concentration of the labeled protein was evaluated by measuring light absorbance at 280 nm. The labeling yield was 80–100%. The success of the labeling reactions was confirmed by mass spectrometry.

### 4.9 EPR spectroscopy

X‐band room temperature (298 K) continuous wave (CW) EPR measurements were recorded on an Elexsys500 Bruker spectrometer equipped with a Super High Q sensitivity resonator operating at X band (9.9 GHz). The microwave power was 10 mW, the magnetic field modulation amplitude was 0.1 mT, the field sweep was 15 mT, and the receiver gain was 60 dB. All samples were analyzed in quartz capillaries (40 μL sensible volume).

The spin concentration was determined by double integration of the EPR signal obtained under non‐saturating conditions, and the labeling yield was evaluated by comparing the spin concentration with that of a standard solution.

The EPR spectra were simulated using SimLabel (Etienne et al. 2023), a MATLAB graphical user interface based on the EasySpin toolbox (Stoll and Schweiger 2006). Simulations were performed using the “*Slow Motion*” mode of SimLabel, which relies on the EasySpin function “*chili*” for continuous‐wave EPR line‐shape analysis in the slow‐to‐intermediate motional regime. A detailed, step‐by‐step guideline for CW‐EPR spectral simulations using the “*chili*” model (covering parameter selection, fitting strategies, and interpretation) has been published previously (Etienne et al. 2023) and is referenced for readers seeking an in‐depth methodological treatment (https://easyspin.org/easyspin/documentation/userguide_chili.html).

### 4.10 NMR spectroscopy

NMR experiments were carried out at 298 K using 0.2–0.3 mL samples of 0.2–0.3 mM purified U‐^15^N or U‐^13^C,^15^N NDRG1*C, both in the unmodified and phosphorylated forms, in 20 mM HEPES buffer at pH 6.5 or 7.5, containing 150 mM NaCl, 1 mM TCEP, and 10% D_2_O, in 3‐mm NMR tubes. ^1^H‐^15^N HSQC spectra (SW = 12,500 × 4347.826 Hz; TD = 2048 × 256) were collected using a Bruker AVANCE NEO/III spectrometer operating at 28.2 T (1200.73 MHz ^1^H Larmor frequency) and equipped with a 3‐mm triple‐resonance inverse TCI z‐gradient cryoprobe. ^13^C‐detected NMR spectra (CON: SW = 5555.556 × 2840.809 Hz; TD = 1024 × 640; CACO: SW = 5555.556 × 6369.427 Hz; TD = 1024 × 350) were acquired using a Bruker AVANCE NEO spectrometer operating at 16.4 T (700.06 MHz ^1^H Larmor frequency), equipped with a 3‐mm TXO cryoprobe optimized for ^13^C direct detection. Proton chemical shifts were referenced to 2,2‐dimethyl‐2‐silapentane‐5‐sulfonic acid sodium salt (DSS), while the ^13^C and ^15^N chemical shifts were referenced indirectly to DSS, using the ratios of the gyromagnetic constants. All NMR spectra were processed using the NMRpipe (Delaglio et al. 1995) software (squared cosine for apodization in all dimensions) and employing the SMILE (Ying et al. 2017) (Sparse Multidimensional Iterative Lineshape‐Enhanced) reconstruction algorithm implemented within the program. Data analysis and resonance assignment were performed using POKY (Lee et al. 2021).

## AUTHOR CONTRIBUTIONS

**Noemi Carosella:** Investigation; validation; formal analysis; writing – review and editing; methodology. **Chiara Pastorello:** Methodology; investigation; formal analysis; writing – review and editing. **Jehan Waeytens:** Methodology; investigation; validation; writing – review and editing; formal analysis; resources. **Ylenia Beniamino:** Methodology; investigation; validation; project administration; writing – review and editing. **Valentina Roncassaglia:** Methodology; investigation; validation. **Lucrezia Serra:** Methodology; investigation; validation; writing – review and editing. **Vincent Raussens:** Validation; formal analysis; supervision; resources; writing – review and editing. **Elisabetta Mileo:** Investigation; validation; formal analysis; supervision; writing – review and editing; resources. **Stefano Ciurli:** Conceptualization; investigation; validation; formal analysis; funding acquisition; writing – review and editing; resources. **Barbara Zambelli:** Conceptualization; methodology; investigation; validation; formal analysis; supervision; funding acquisition; resources; writing – original draft; writing – review and editing.

## CONFLICT OF INTEREST STATEMENT

The authors declare no conflicts of interest.

## Supporting information


**Data S1.** Supporting Information.

## Data Availability

The data that support the findings of this study are available from the corresponding author upon reasonable request.
