# Supplementary material for: Phosphorylation disrupts the interaction between the intrinsically disordered region of the oncogenic NDRG1 and lipid vesicles
Source: Protein Sci. 2026 Feb 22;35(3):e70510. doi: 10.1002/pro.70510 (PMC12928042; doi:10.1002/pro.70510)
Supplement: Supplementary file 1 — Data S1. Supporting Information. [file PRO-35-e70510-s001.docx]

**Phosphorylation disrupts the interaction between the intrinsically disordered region of the onogenic NDRG1 and lipid vescicles**

Noemi Carosella^a^, Chiara Pastorello^b,c^, Jehan Waeytens^d^, Ylenia Beniamino^a^, Valentina Roncassaglia^a^, Lucrezia Serra^a,e^, Vincent Raussens^f^, Elisabetta Mileo^b^, Stefano Ciurli^a^, Barbara Zambelli^a*^

^a^Laboratory of Bioinorganic Chemistry, Department of Pharmacy and Biotechnology (FaBiT), University of Bologna, Italy

^b^Aix-Marseille Univ, CNRS,BIP, Bioénergétique et Ingénierie des Protéines, Marseille, France.

^c^Aix-Marseille Univ, CNRS, INSERM, Institut Paoli-Calmettes, CRCM, Marseille, France.

^d^RD3-Pharmacognosy, Bioanalysis and Drug Discovery Unit, Faculty of Pharmacy, Université Libre de Bruxelles, B-1050 Bruxelles, Belgium

^e^IRCCS Istituto delle Scienze Neurologiche di Bologna, Italy (current address)

^f^Structure et Fonction des Membranes Biologiques, Université libre de Bruxelles, B-1050 Bruxelles, Belgium

**SUPPLEMENTARY INFORMATION**

**
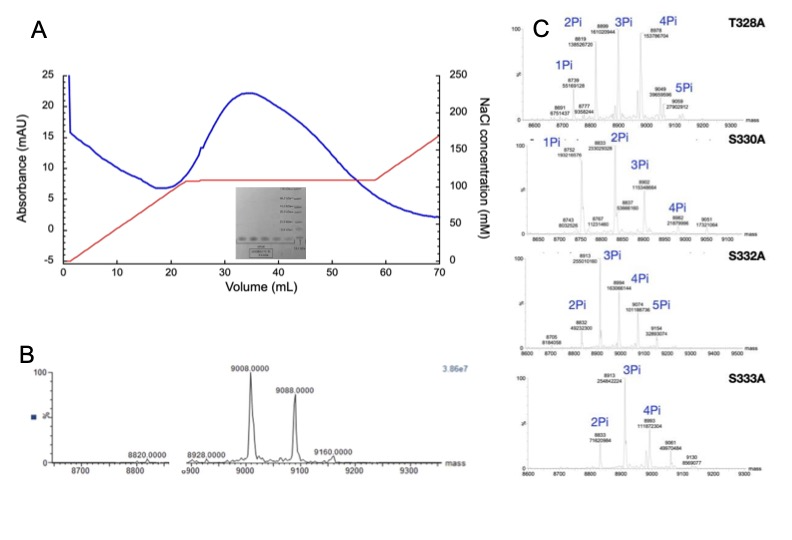
**

**Figure SI-1.** (A) Cation exchange chromatography elution profile of phosphorylated NDRG1*C (P-NDRG1*C). Fractions corresponding to the eluted peak are shown in the SDS-PAGE gel. (B) Mass spectrometry profile of the P-NDRG1*C. Molar mass of unmodified NDRG1*C is 8,689 Da. The presence of a phosphate group increases the molar mass of the protein by 79 Da for each deprotonated phosphate, indicating that the samples contains both tetra- and penta-phosphorylated NDRG1*C in similar amounts. (C) Mass spectrometry profiles of the P-NDRG1*C mutants. Molar masses for unmodified Ser-to-Ala mutant and Thr-to-Ala mutant are 8,673 Da and 8,659 Da, respectively. Peaks corresponding to phosphorylated protein are indicated.


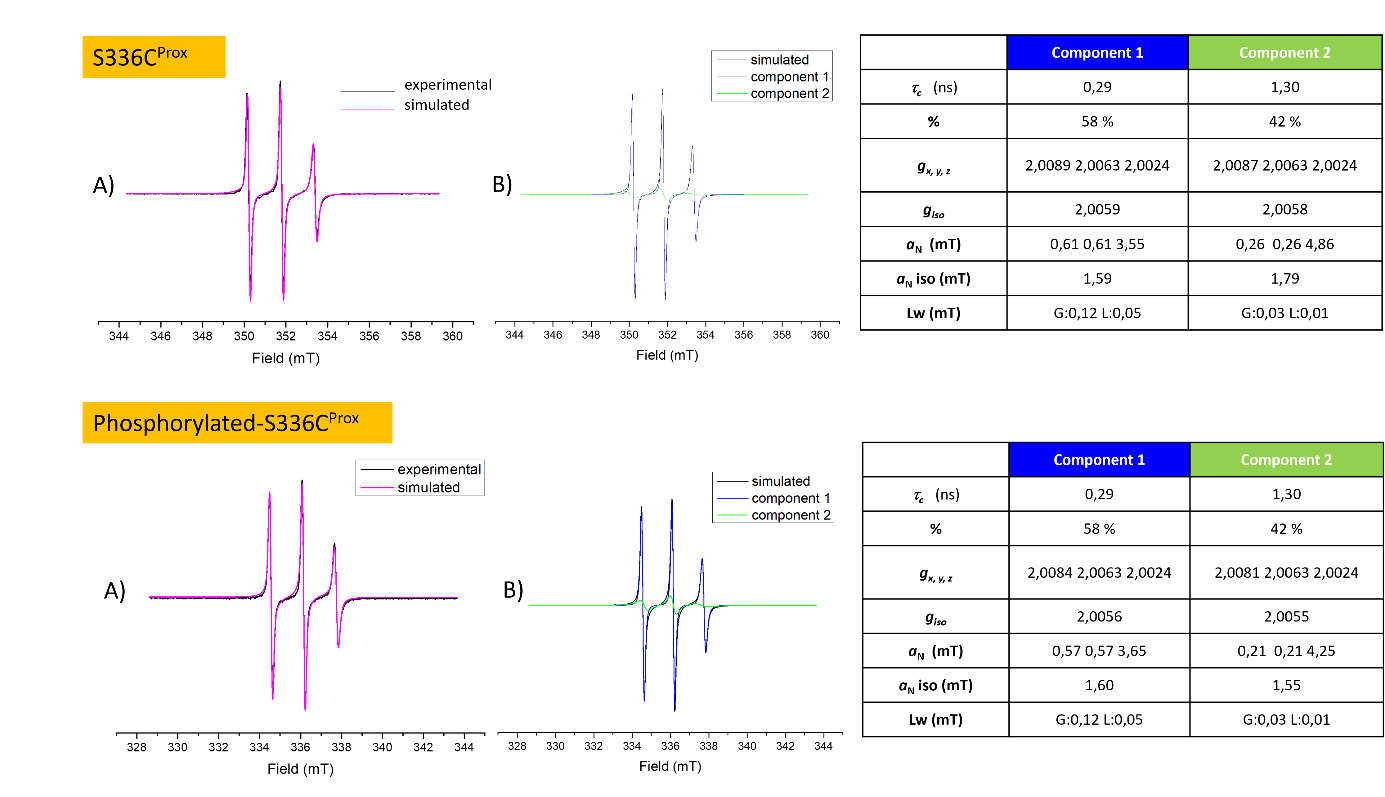


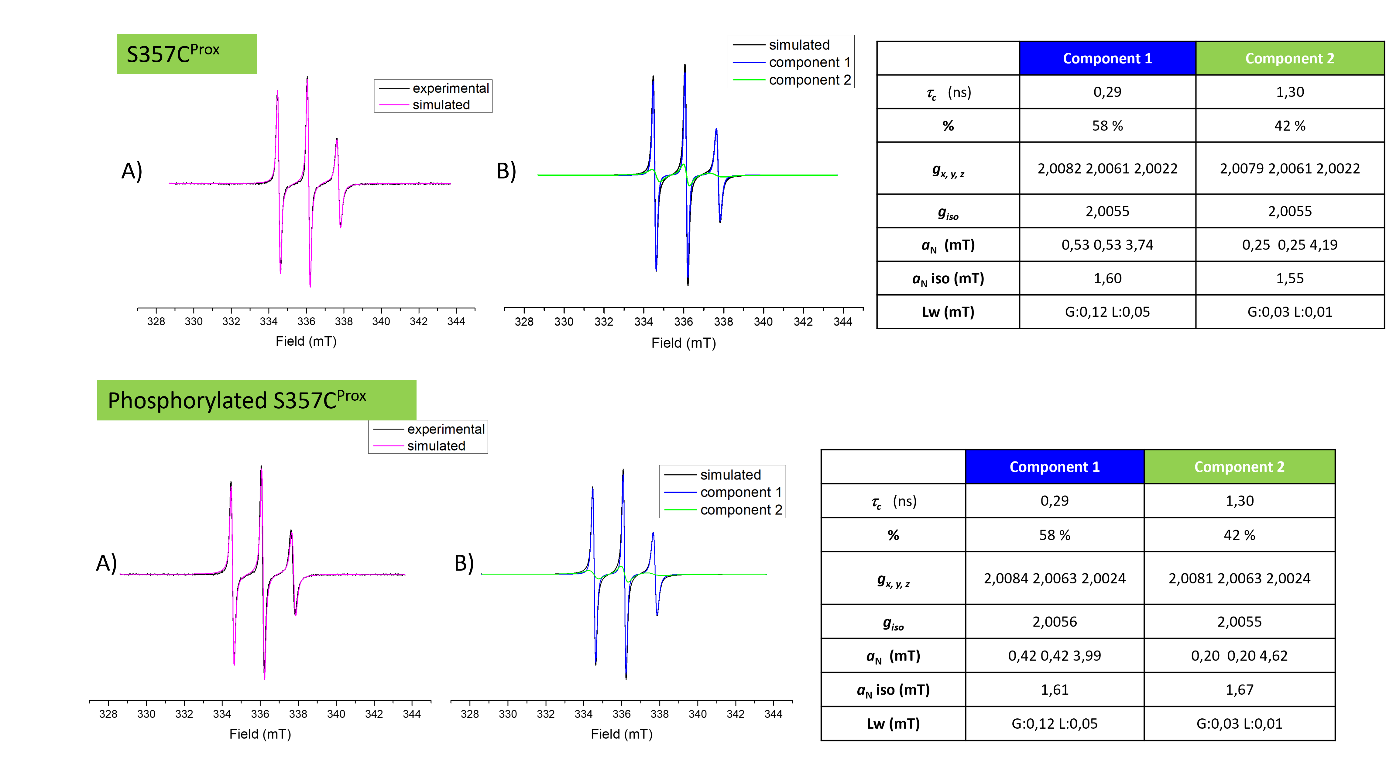


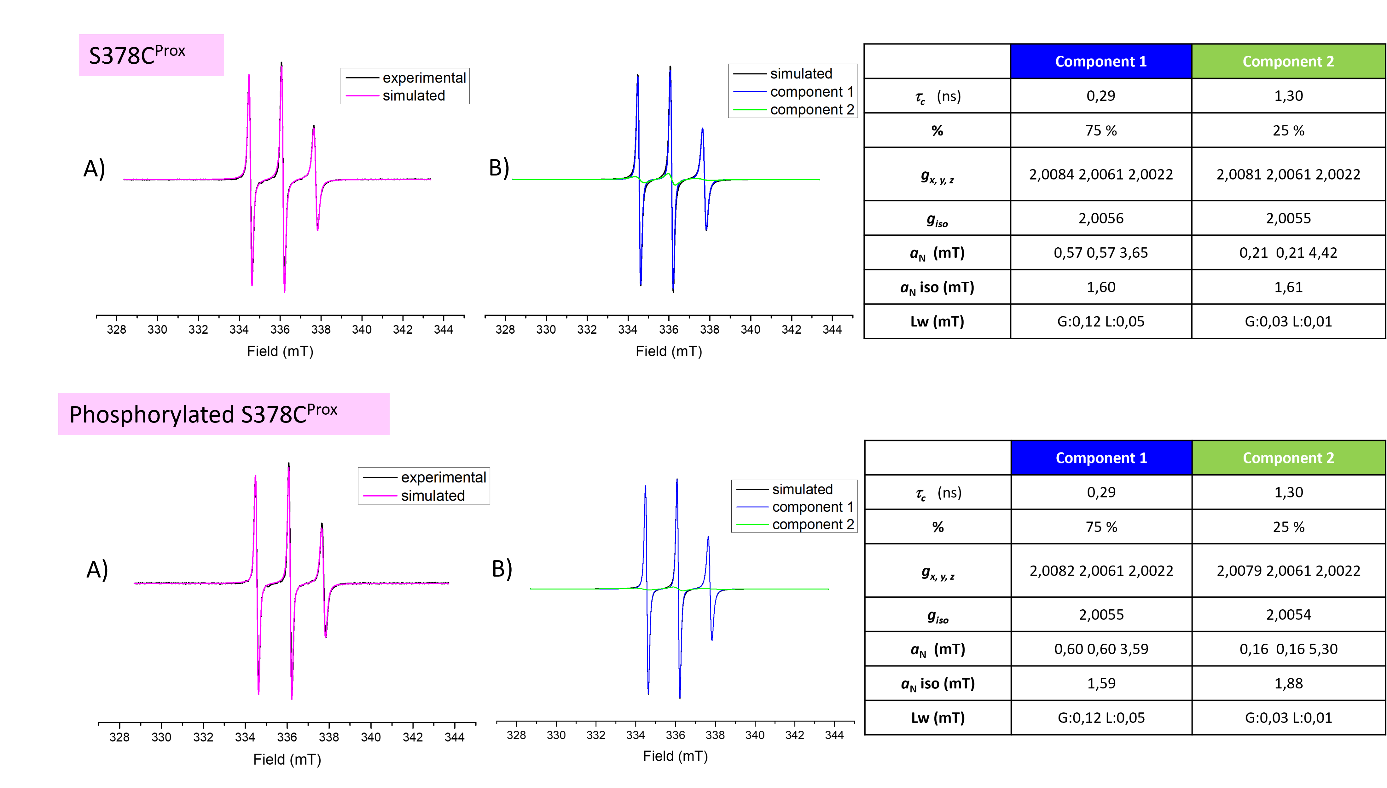


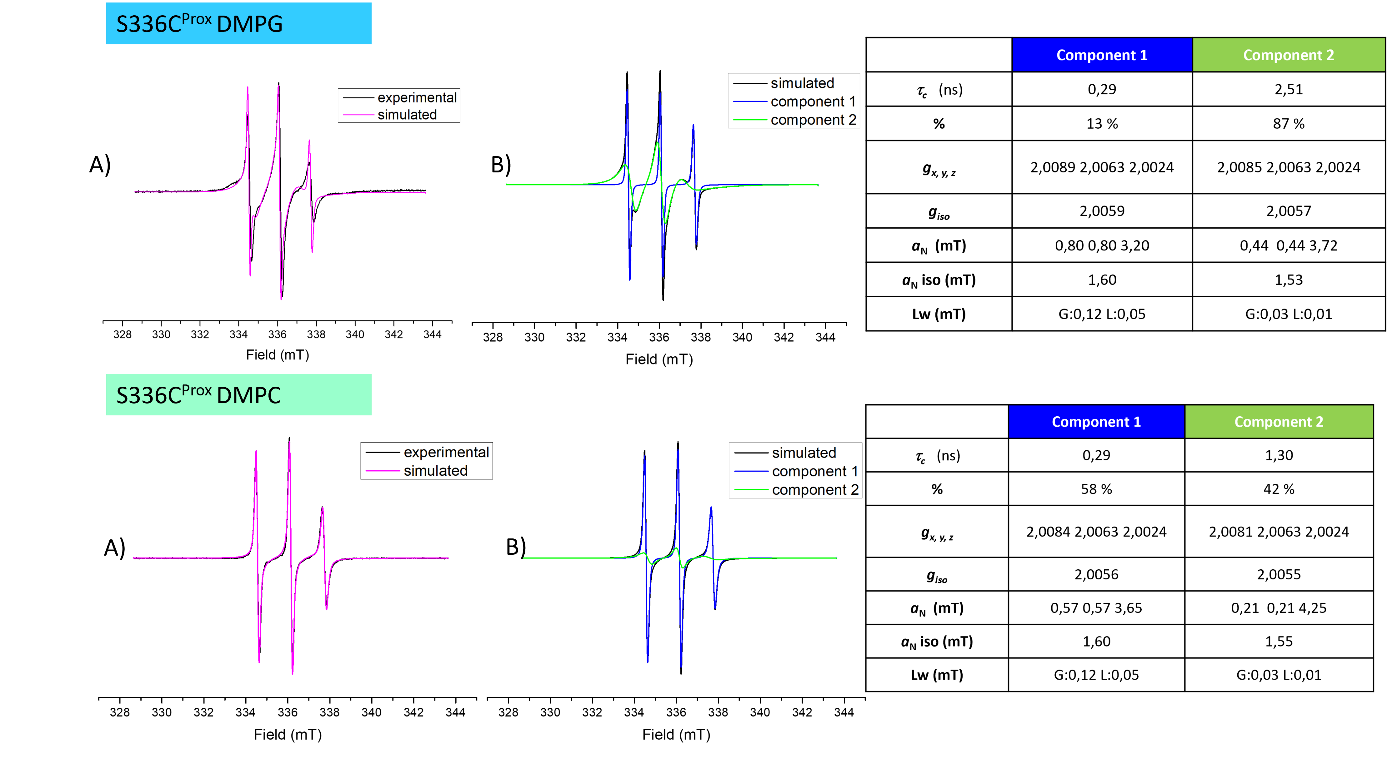


**Figure SI-2.** Experimental EPR spectra of NDRG1*C variants labeled with spin label Maleimido-Proxyl. Simulations were performed with the ‘Slow Motion’ mode of SimLabel program^2^ (a GUI of EasySpin^3^), while the used routine is described here^2^. The EasySpin function used for spectra simulation was ‘chili’. All panels “A” show the simulated spectra (*magenta line*) superimposed on the experimental ones (*black line*). All panels “B” show the components required to obtain the best fits. The tables on the right side indicate all parameters used for the simulations.


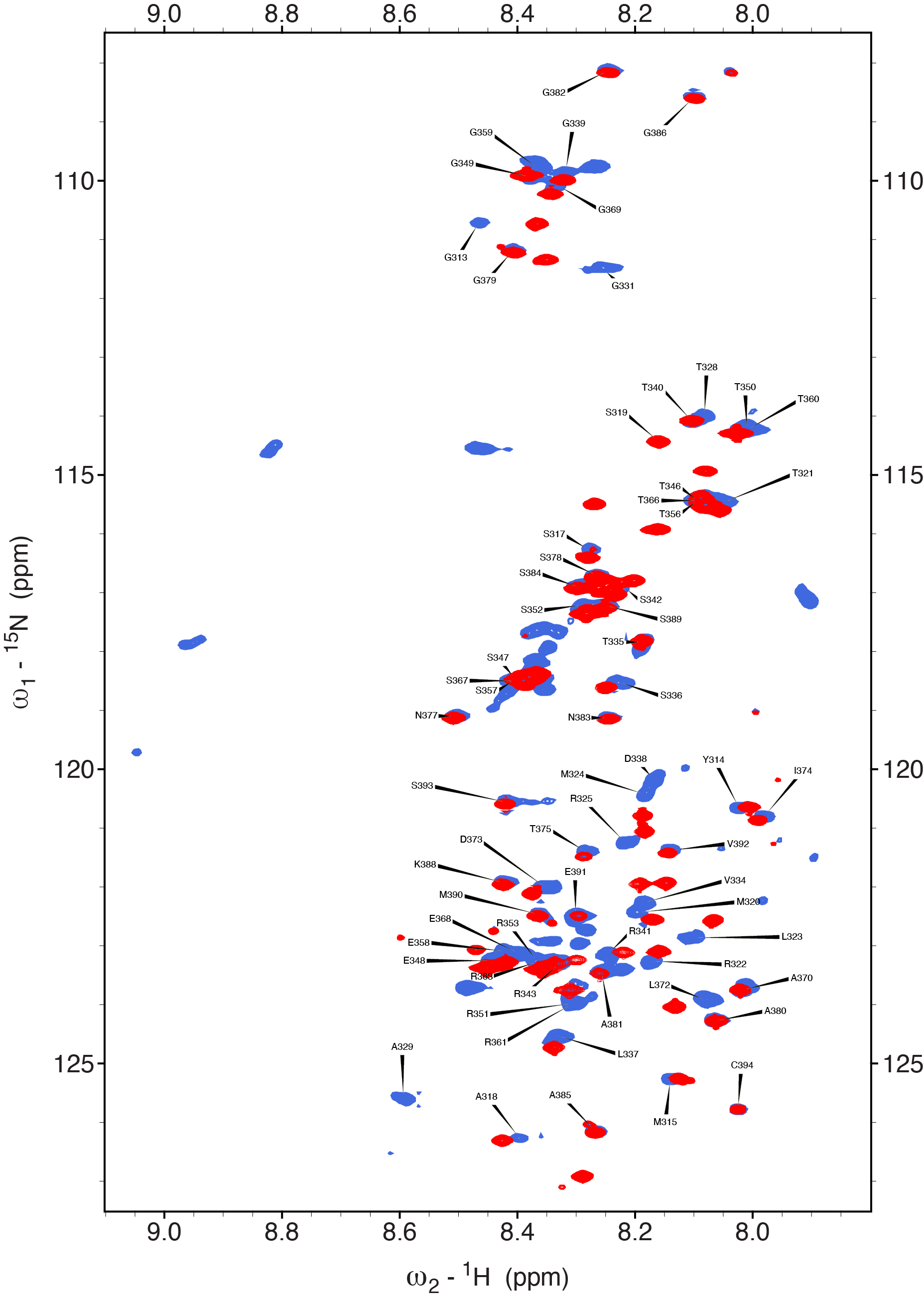


**Figure SI-3.** Overlay of ^1^H-^15^N HSQC NMR spectra of NDRG1*C in the unmodified (red) and phosphorylated (blue) state at pH 6.5.


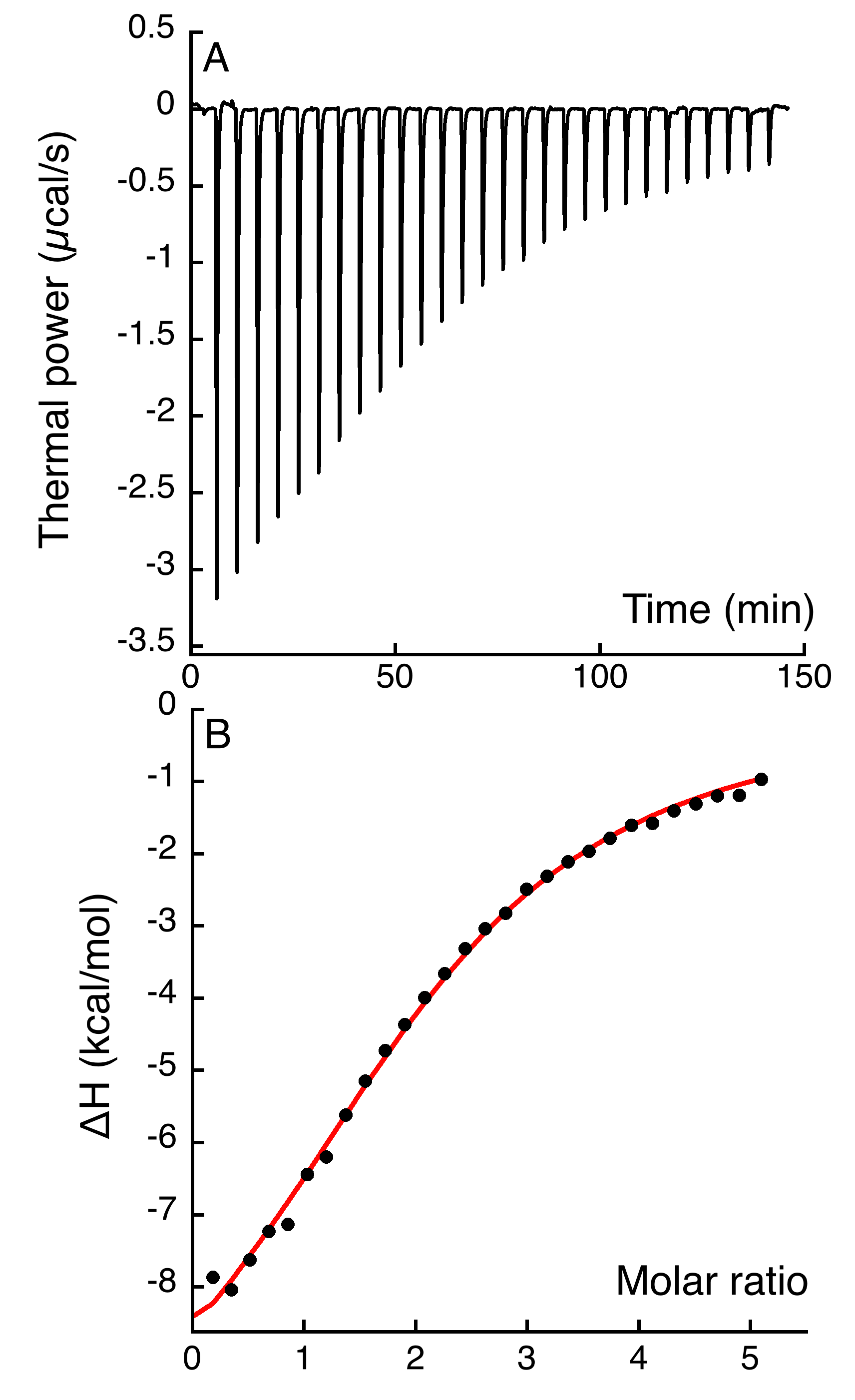


**Figure SI-4.** Ni(II) titration over NDRG1*C by ITC, under conditions previously reported^1^. Upper panel represents raw data for injections of Ni(II) solution into protein solution. The bottom panel reports the integrated data (filled circles) and the fit of the experimental data (red line).

**
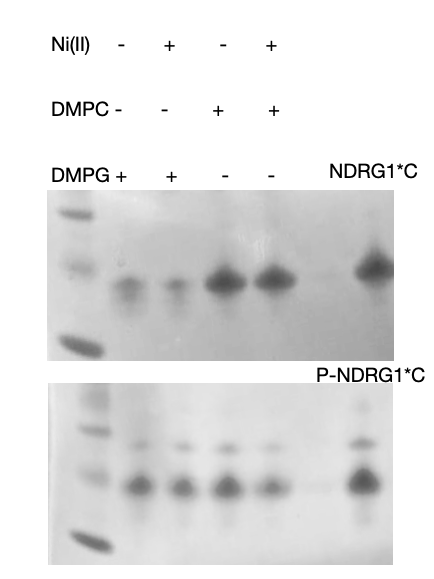
**

**Figure SI-5.** Supernatants deriving from co-sedimentation analysis of unmodified and phosphorylated NDRG1*C and liposomes with different lipid composition, in the absence and in the presence of two equivalents of Ni(II).


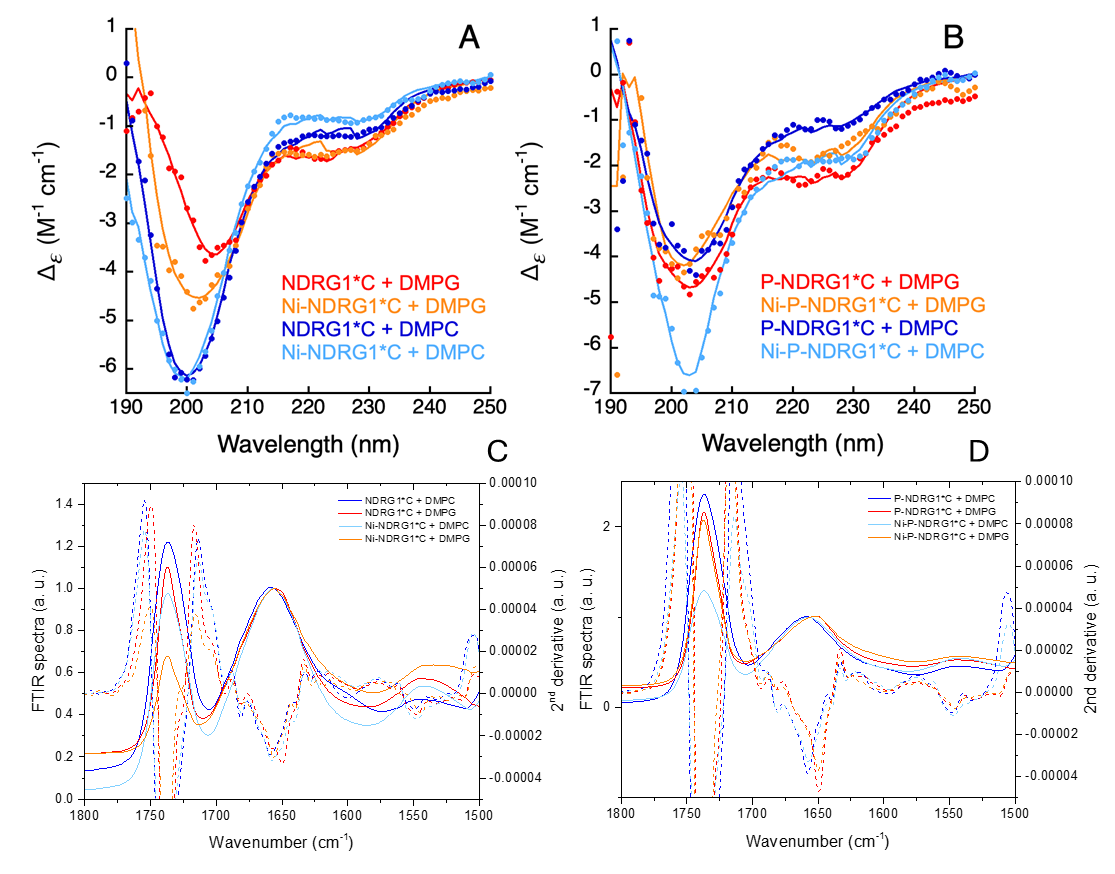


**Figure SI-6**. Circular dichroism of NDRG1*C (A) and P-NDRG1*C (B) and FTIR analysis of NDRG1*C (C) and P-NDRG1*C (D) in the presence of DMPC (blue), Ni(II) and DMPC (cyan), DMPG (red) and DMPG and Ni(II) (orange). For CD spectra, filled circles represent experimental points, solid lines are the fits obtained using BestSel. The FTIR spectra are represented by solid lines and 2^nd^ derivatives by dashed lines.


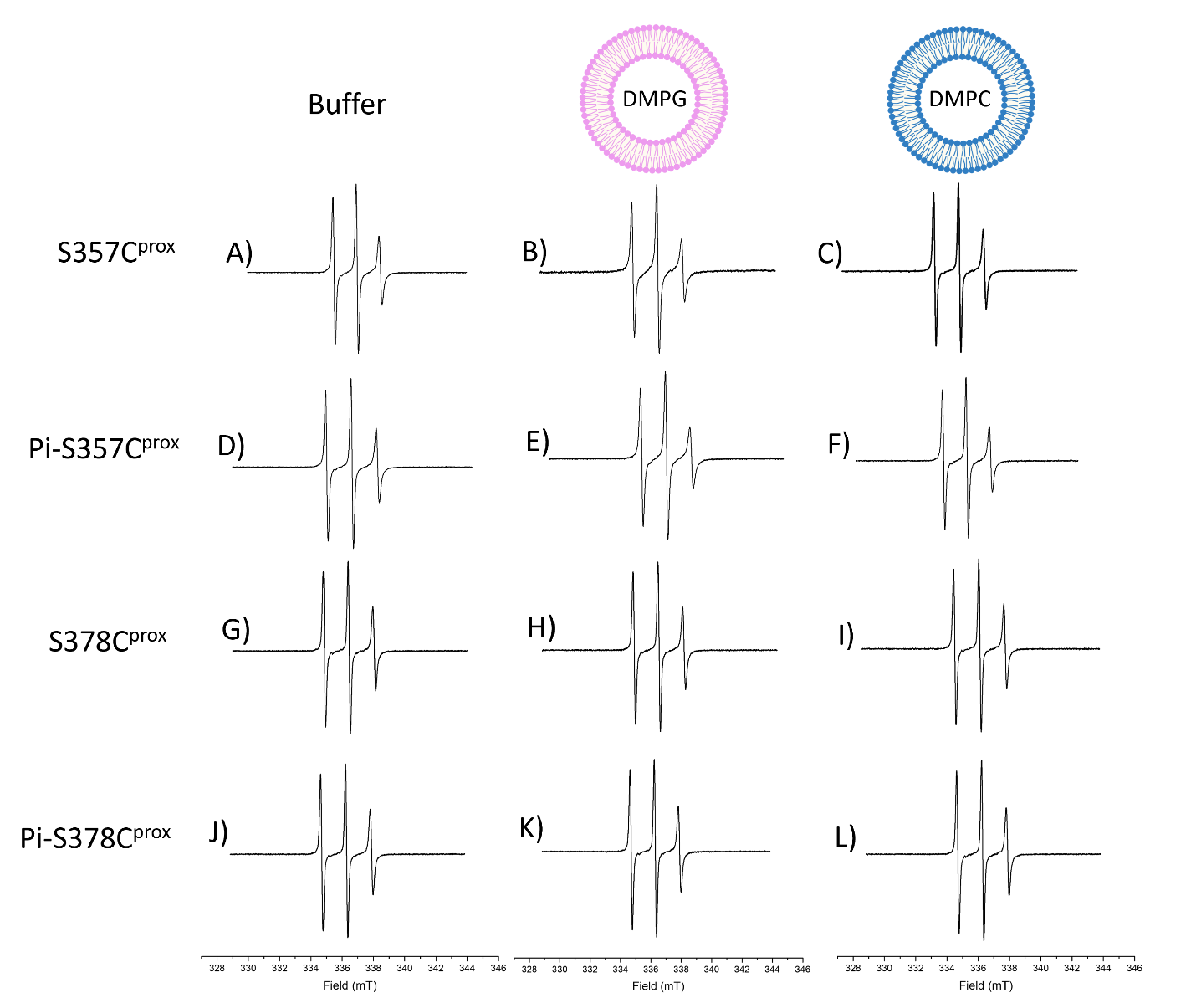


**Figure SI-7**- X-Band CW room temperature EPR spectra in buffer solution for variant S357C^prox^ (A), Pi-S357C^prox^ (D), S378C^prox^ (G), Pi-S378C^prox^ (J); in the presence of DMPG LUVs for variant S357C^prox^ (B), Pi-S357C^prox^ (E), S378C^prox^ (H), Pi-S378C^prox^ (K); in the presence of DMPC LUVs for variant S357C^prox^ (C), Pi-S357C^prox^ (F), S378C^prox^ (I), Pi-S378C^prox^ (L).


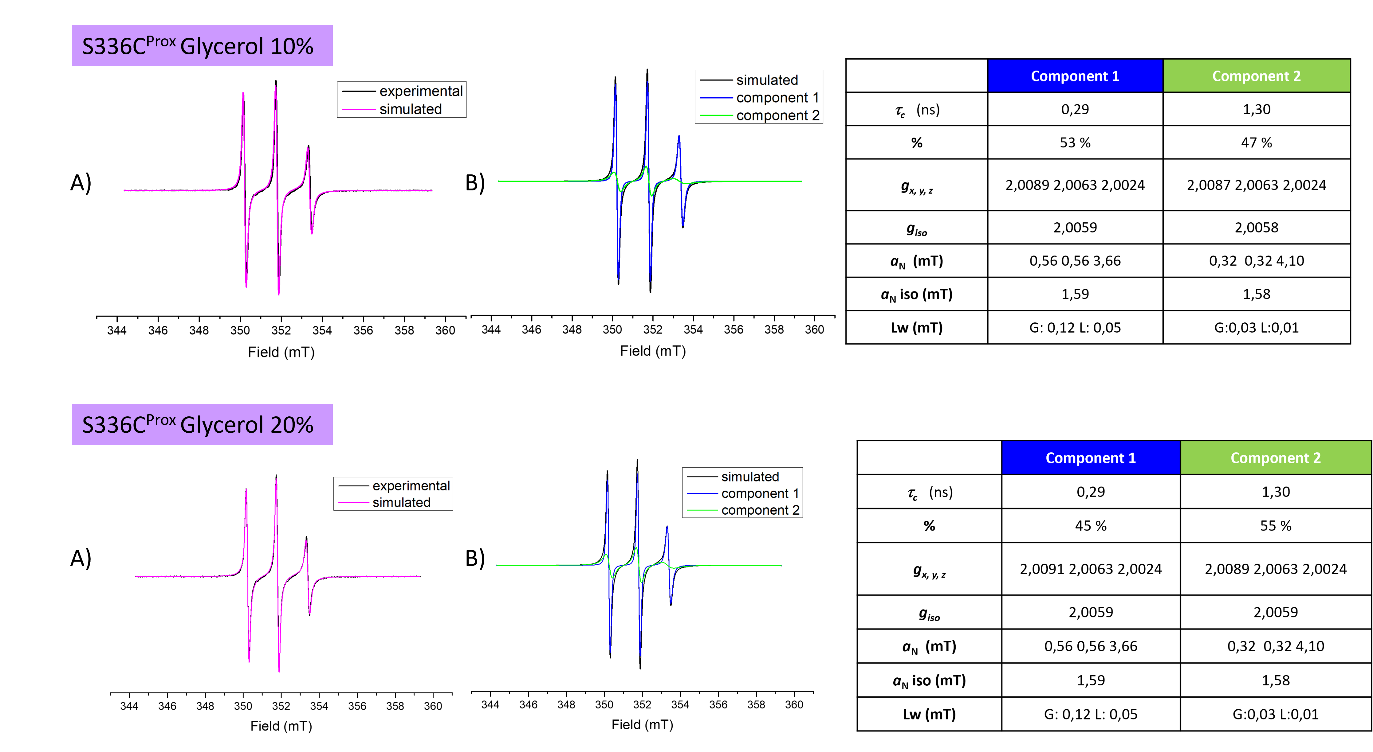


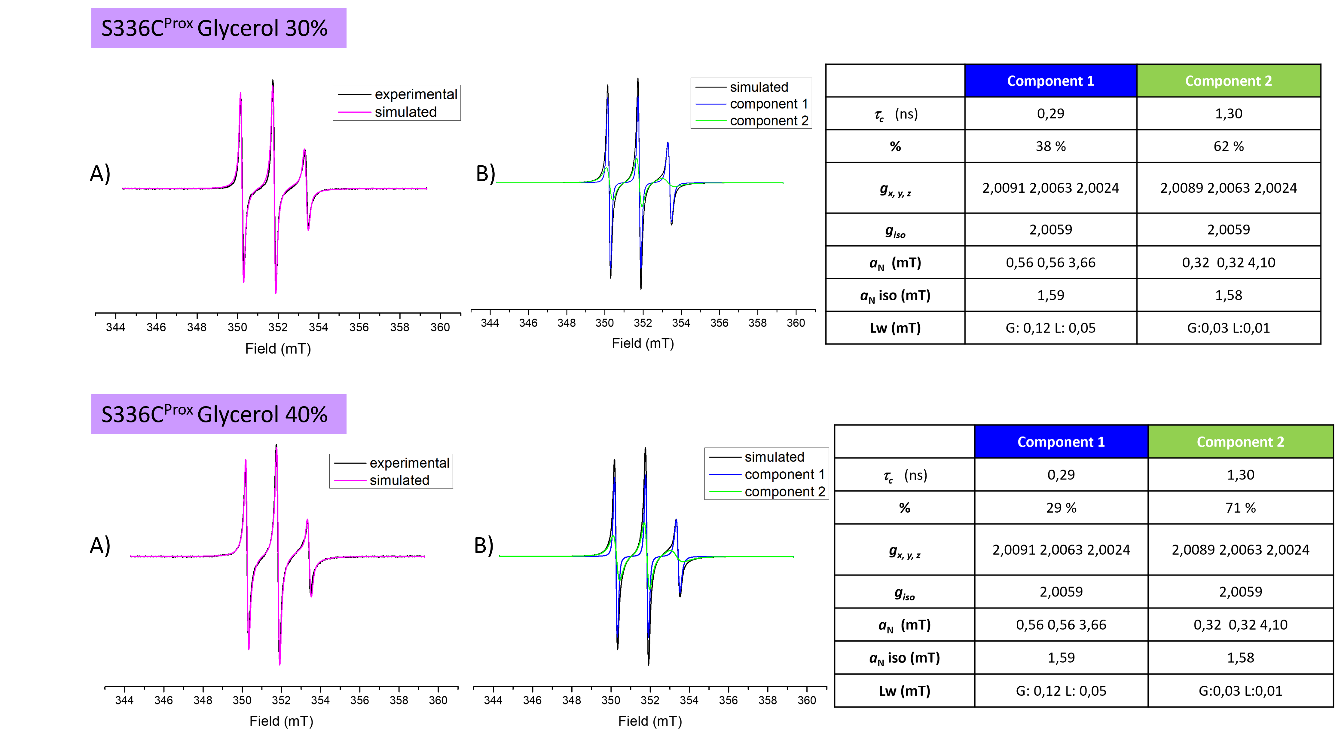


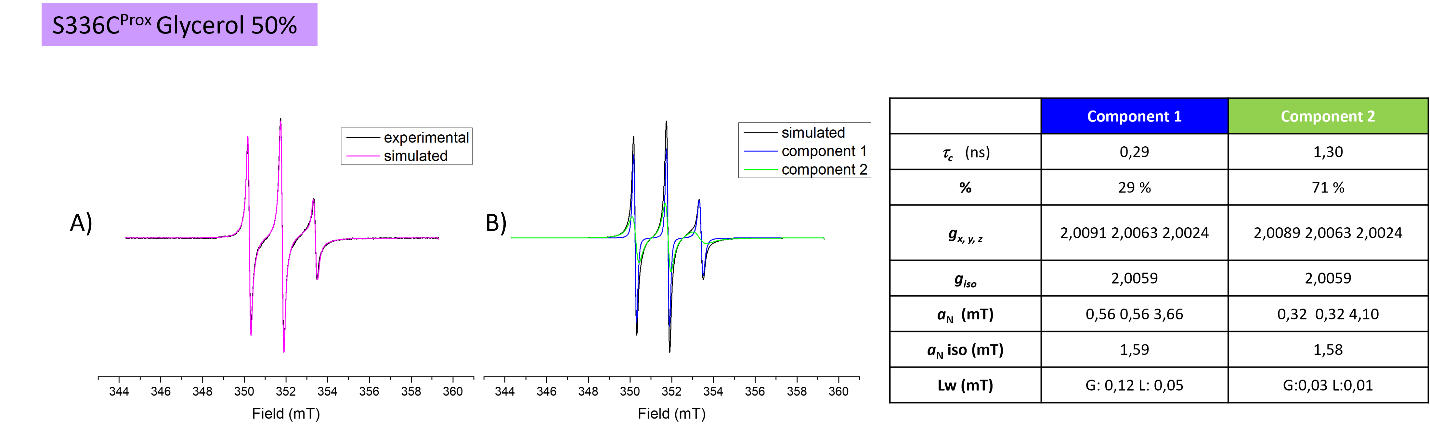


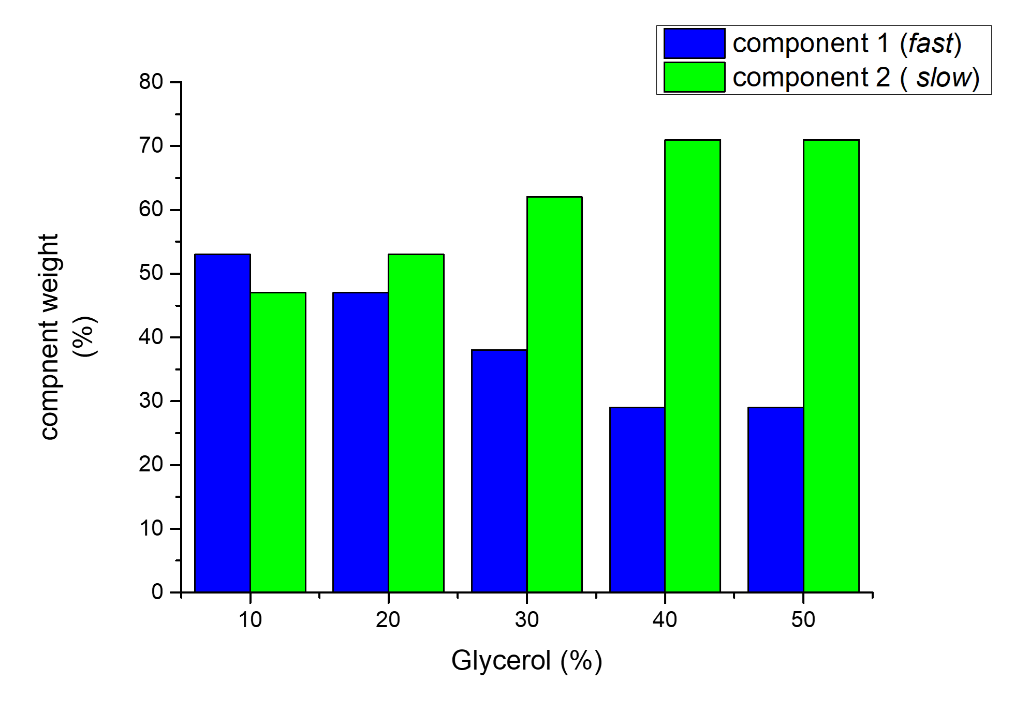


**Figure SI-8** – Experimental EPR spectra of NDRG1*C variants labeled with Maleimido-Proxyl in the presence of different amounts of glycerol. Simulations were performed with the ‘Slow Motion’ mode of SimLabel program^1^ (a GUI of EasySpin^2^) using a previously described protocol.^1^ The EasySpin function used for spectra simulation was ‘chili’. All panels “A” show the simulated spectra (magenta line) superimposed on the experimental ones (black line). All panels “B” show the components required to obtain the best fits. The tables on the right side report the parameters used for simulations. In the bottom plot, the variation in component weights is plotted as a function of the glycerol content

**Figure SI-9.** Intensity ratios of the peaks in the CACO spectra obtained at 16.4 T by ^13^C direct detection at pH 7.5 for NDRG1*C in the absence and presence of DMPC vesicles.


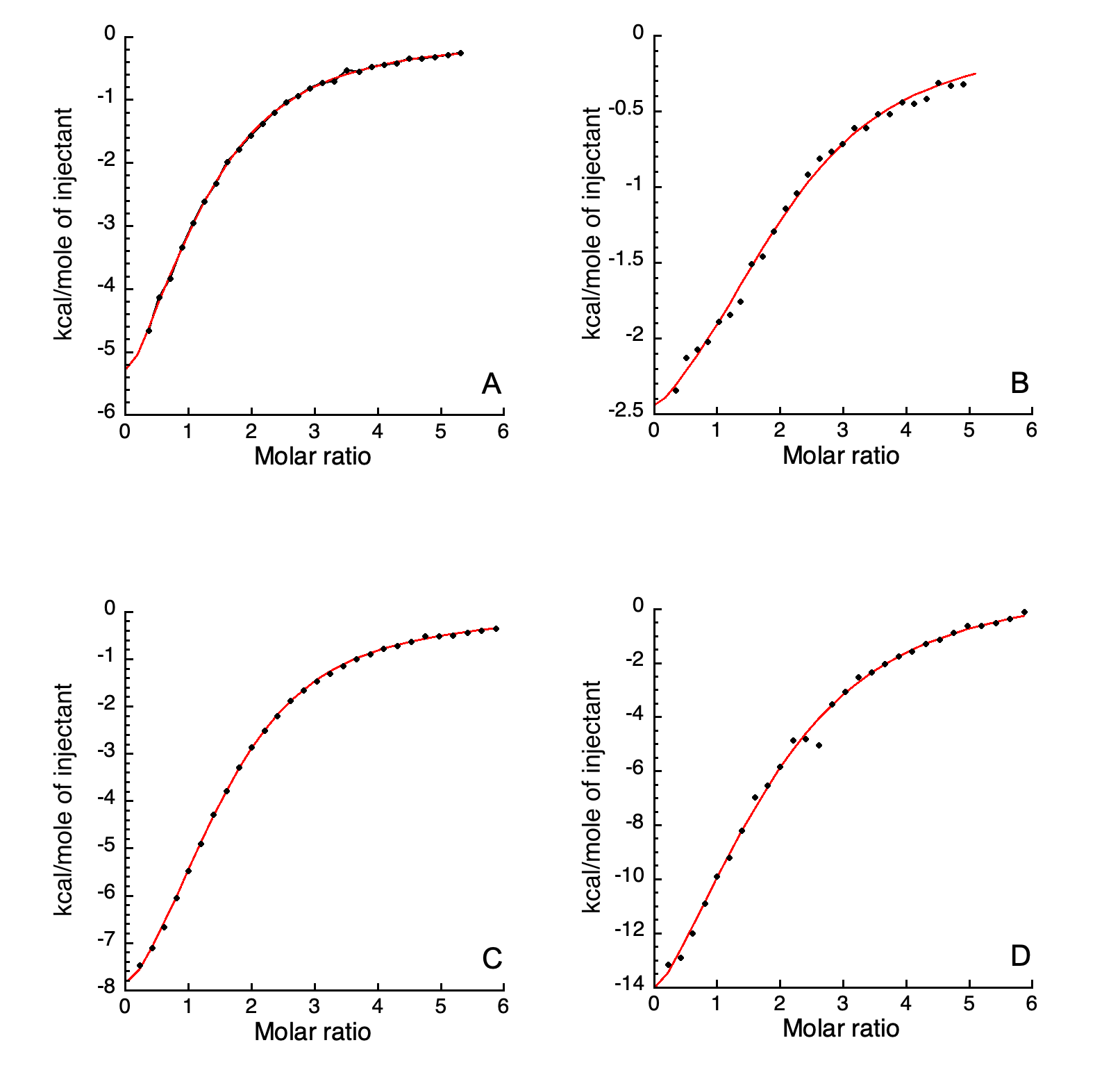


**Figure SI-10.** Ni(II) titration over NDRG1*C by ITC under conditions previously reported^1^, in its unmodified (A, B) and phosphorylated states (C, D) and in the presence of DMPC (A, C) and DMPG (B, D). The figures show integrated data (filled circles) and the fit of the experimental data (red line).

**Table SI-1.** Assignment of the amide I band of protein by FTIR

| **Secondary structure** | **Band position in H_2_O (cm^-1^)** | |
| --- | --- | --- |
|  | *Center* | *Range* |
| α-helix | 1654 | 1648–1657 |
| β-sheet | 1633 | 1623–1641 |
|  | 1684 | 1674–1695 |
| Turns | 1672 | 1662–1686 |
| Disordered | 1654 | 1642–1657 |

**Table SI-2.** Thermodynamic parameters of Ni(II) binding derived from isothermal titration calorimetry experiments

| **Protein samples** | ***K_A_*** | ***K_D_* (µM)** | ***ΔH* (kcal/mol)** | ***ΔS* (cal mol^-1^ K^-1^)** | ***ΔG* (kcal/mol)** |
| --- | --- | --- | --- | --- | --- |
| NDRG1*C | 3.0 ± 0.2 x 10^4^ | 34 ± 2 | -11.6 ± 0.3 | -18.3 | -6.10 ± 0.07 |
| P-NDRG1*C | 7.5 ± 0.4 x 10^4^ | 13.3 ± 0.7 | -11.9 ± 0.5 | -17.5 | -6.65 ± 0.05 |
| NDRG1*C + DMPC | 4.2 ± 0.1 x 10^4^ | 23.6 ± 0.6 | -8.5 ± 0.2 | -7.41 | -6.31 ± 0.02 |
| NDRG1*C+DMPG | 3.90 ± 0.4 x 10^4^ | 26 ± 3 | -3.2 ± 0.1 | +10.0 | -6.3 ± 0.1 |
| P-NDRG1*C + DMPC | 5.8 ± 0.1 x 10^4^ | 17.2 ± 0.3 | -10.8 ± 0.1 | -14.3 | -6.50 ± 0.02 |
| P-NDRG1*C + DMPG | 3.5 ± 0.3 x 10^4^ | 29 ± 2 | -23 ± 1 | -56.3 | -6.19 ± 0.09 |

**References**

(1) Beniamino, Y.; Cenni, V.; Piccioli, M.; Ciurli, S.; Barbara Zambelli. The Ni(II)-Binding Activity of the Intrinsically Disordered Region of Human NDRG1, a Protein Involved in Cancer Development. *Biomolecules* **2022**, *12* (9), 1272. https://doi.org/10.3390/biom12091272.

(2) Etienne, E.; Pierro, A.; Tamburrini, K. C.; Bonucci, A.; Mileo, E.; Martinho, M.; Belle, V. Guidelines for the Simulations of Nitroxide X-Band Cw EPR Spectra from Site-Directed Spin Labeling Experiments Using SimLabel. *Molecules* **2023**, *28* (3), 1348. https://doi.org/10.3390/molecules28031348.

(3) Stoll, S.; Schweiger, A. EasySpin, a Comprehensive Software Package for Spectral Simulation and Analysis in EPR. *J. Magn. Reson.* **2006**, *178* (1), 42–55. https://doi.org/10.1016/j.jmr.2005.08.013.
